# β-Cell Dysfunction in COVID-19 and Post-COVID Syndrome: Molecular Mechanisms Linking Inflammation, Oxidative Stress, and Insulin Secretion

**DOI:** 10.3390/ijms27167083

**Published:** 2026-08-07

**Authors:** Victoria Tsvetkova, Katya Todorova

**Affiliations:** 1 Department of Cardiology, Pulmonology and Endocrinology, Medical University, 5800 Pleven, Bulgaria; 2 Clinic of Endocrinology and Metabolic Disease, University Hospital Georgi Stranski, 5800 Pleven, Bulgaria

**Keywords:** COVID-19, SARS-CoV-2, β-cell dysfunction, insulin secretion, post-COVID syndrome, oxidative stress, mitochondrial dysfunction, hypoxia, insulin resistance, new-onset diabetes

## Abstract

Coronavirus disease 2019 (COVID-19) is increasingly recognized as a multisystem disorder associated with persistent metabolic complications extending beyond the acute phase of infection. Accumulating evidence suggests that severe acute respiratory syndrome coronavirus 2 (SARS-CoV-2) may disrupt glucose homeostasis through mechanisms involving pancreatic β-cell dysfunction, insulin resistance, chronic inflammation, oxidative stress, mitochondrial dysfunction, and hypoxia-related signalling. This review summarizes current evidence regarding the molecular and cellular mechanisms linking SARS-CoV-2 infection to impaired insulin secretion and post-COVID metabolic disturbances. Particular emphasis is placed on the regulation of insulin secretion, β-cell compensation and failure, oxidative stress, inflammatory signalling, mitochondrial dysfunction, and the development of the post-COVID metabolic phenotype. Emerging evidence indicates that persistent metabolic abnormalities after COVID-19 may range from transient dysglycaemia to new-onset diabetes mellitus and metabolic syndrome. The review also discusses clinical implications, biomarkers, therapeutic perspectives, and unresolved questions regarding the reversibility of post-COVID β-cell dysfunction. A better understanding of the mechanisms underlying post-COVID metabolic dysfunction may improve risk stratification, facilitate early intervention, and support development of targeted therapeutic strategies aimed at preserving β-cell function and long-term metabolic health.

## 1. Introduction

Since its emergence in late 2019, severe acute respiratory syndrome coronavirus 2 (SARS-CoV-2) has become a global health challenge with profound multisystem consequences. Although coronavirus disease 2019 (COVID-19) was initially regarded primarily as an acute respiratory illness, accumulating clinical and experimental evidence has demonstrated that SARS-CoV-2 can affect multiple organ systems, including the cardiovascular, neurological, renal, endocrine, and metabolic systems [1,2,3]. Recognition of persistent symptoms and organ dysfunction following acute infection has shifted scientific attention towards the long-term biological consequences of SARS-CoV-2 infection, collectively termed post-COVID condition (Long COVID), which is now recognised as a complex multisystem disorder rather than a single clinical entity [4,5].

Among the long-term sequelae of COVID-19, disturbances in metabolic homeostasis have emerged as an important clinical concern. Persistent abnormalities in glucose metabolism, insulin sensitivity, and energy homeostasis have been described across diverse patient populations, indicating that metabolically active tissues—including the pancreas, adipose tissue, liver, skeletal muscle, and vascular endothelium—remain functionally affected long after resolution of the acute infection [6,7,8,9,10,11,12]. These observations have raised increasing concern regarding the potential contribution of COVID-19 to the future burden of diabetes mellitus and cardiometabolic disease while prompting questions as to whether these abnormalities represent transient consequences of acute illness or define a distinct post-infectious metabolic disorder.

The relationship between COVID-19 and metabolic disease is increasingly recognised as bidirectional. Pre-existing obesity, diabetes mellitus, and metabolic syndrome substantially increase the risk of severe COVID-19 and adverse clinical outcomes [6,7,8]. Conversely, SARS-CoV-2 infection has been associated with persistent insulin resistance, impaired glucose tolerance, β-cell dysfunction, dysglycaemia, and an increased incidence of newly diagnosed diabetes mellitus in individuals without previously recognised metabolic disease [9,10,11,12,13,14,15,16,17]. However, the mechanisms responsible for these metabolic abnormalities remain incompletely understood and are likely to extend beyond the direct consequences of acute viral infection.

Growing evidence suggests that pancreatic β-cell dysfunction represents a central component of post-COVID metabolic abnormalities. Nevertheless, β-cell impairment should not be considered an isolated phenomenon but rather part of a broader network of interacting biological processes involving chronic inflammation, immune dysregulation, oxidative stress, mitochondrial dysfunction, endoplasmic reticulum stress, endothelial injury, and altered interorgan communication. The relative contribution of these mechanisms probably varies among individuals, contributing to the marked heterogeneity observed in clinical presentation and long-term metabolic outcomes [13,14,15,16,17,18].

Several key aspects of disease pathogenesis remain controversial. Although numerous experimental studies have demonstrated that SARS-CoV-2 can impair pancreatic β-cell function, the extent to which this reflects direct viral infection versus indirect injury mediated by systemic inflammation, metabolic stress, endothelial dysfunction, or persistent immune activation remains unresolved. Likewise, growing evidence supporting viral persistence in extrapulmonary tissues has generated considerable interest as a potential driver of chronic inflammation and sustained metabolic dysfunction, although its precise contribution continues to be actively debated [5,15,16,17]. These unresolved questions highlight the complexity of post-COVID metabolic disease and emphasise the need for integrative mechanistic models capable of explaining its biological heterogeneity.

In this review, the term post-COVID metabolic phenotype refers to the heterogeneous spectrum of persistent metabolic abnormalities developing after SARS-CoV-2 infection, including impaired insulin secretion, insulin resistance, dysglycaemia, new-onset diabetes mellitus, metabolic syndrome, or combinations of these conditions. Rather than representing a single disease entity, this phenotype encompasses multiple biological trajectories arising from differences in host susceptibility, disease severity, and the dynamic interaction of molecular and cellular mechanisms. Recognition of this heterogeneity provides an essential framework for understanding the diverse pathogenic pathways discussed throughout this review.

Although numerous reviews have examined diabetes, insulin resistance, or metabolic complications associated with COVID-19 [5,9,17], many have focused predominantly on individual pathogenic mechanisms rather than integrating the complex interactions among β-cell dysfunction, immune dysregulation, oxidative stress, mitochondrial impairment, endothelial injury, viral persistence, and systemic metabolic remodelling into a unified mechanistic framework. Consequently, current understanding of post-COVID metabolic dysfunction remains fragmented despite the rapidly expanding body of experimental and clinical evidence.

Against this background, the present narrative review aims to provide an integrated overview of the molecular mechanisms underlying pancreatic β-cell dysfunction following SARS-CoV-2 infection and their contribution to the development of the post-COVID metabolic phenotype. Particular emphasis is placed on the interplay between direct and indirect mechanisms of β-cell injury, intracellular pathways regulating insulin secretion and β-cell survival, immune and vascular mechanisms, emerging biomarkers, and future precision medicine approaches. By integrating molecular, cellular, and clinical evidence, we propose a systems-level framework that may facilitate future mechanistic research while supporting the development of improved strategies for early diagnosis, risk stratification, and targeted therapeutic intervention in post-COVID metabolic disease.

## 2. Literature Search Strategy and Review Methodology

This narrative review summarizes current evidence regarding pancreatic β-cell dysfunction and post-COVID metabolic disturbances associated with severe acute respiratory syndrome coronavirus 2 (SARS-CoV-2) infection.

A structured literature search was performed using the PubMed/MEDLINE, Scopus, and Web of Science databases to identify relevant publications. The primary literature search focused on studies published between January 2020 and July 2026. Earlier landmark publications were additionally included where necessary to provide the physiological and mechanistic background on pancreatic β-cell biology, insulin secretion, mitochondrial function, oxidative stress, endoplasmic reticulum homeostasis, and diabetes pathophysiology.

Search terms included combinations of the following keywords: “COVID-19”, “SARS-CoV-2”, “Long COVID”, “post-COVID condition”, “pancreatic β-cell”, “β-cell dysfunction”, “insulin secretion”, “dysglycaemia”, “new-onset diabetes”, “insulin resistance”, “oxidative stress”, “mitochondrial dysfunction”, “endoplasmic reticulum stress”, “hypoxia”, “hypoxia-inducible factor”, “endothelial dysfunction”, “viral persistence”, “adipose tissue”, “adipokines”, “immune dysregulation”, and “immunometabolism”.

Priority was given to peer-reviewed original experimental studies, clinical investigations, mechanistic studies, systematic reviews, meta-analyses, and international consensus statements directly relevant to pancreatic β-cell function and post-COVID metabolic abnormalities. Additional publications were identified through manual screening of the reference lists of relevant articles.

The available evidence was critically evaluated and integrated to summarize the current understanding of the molecular, cellular, and clinical mechanisms linking SARS-CoV-2 infection to persistent β-cell dysfunction and metabolic disease. Particular emphasis was placed on the interactions among inflammation, oxidative stress, mitochondrial dysfunction, endoplasmic reticulum stress, endothelial injury, adipose tissue dysfunction, immune dysregulation, and impaired insulin secretion, as well as on emerging biomarkers and potential therapeutic strategies.

No new human or animal studies were performed for this review.

**Figure 1:**
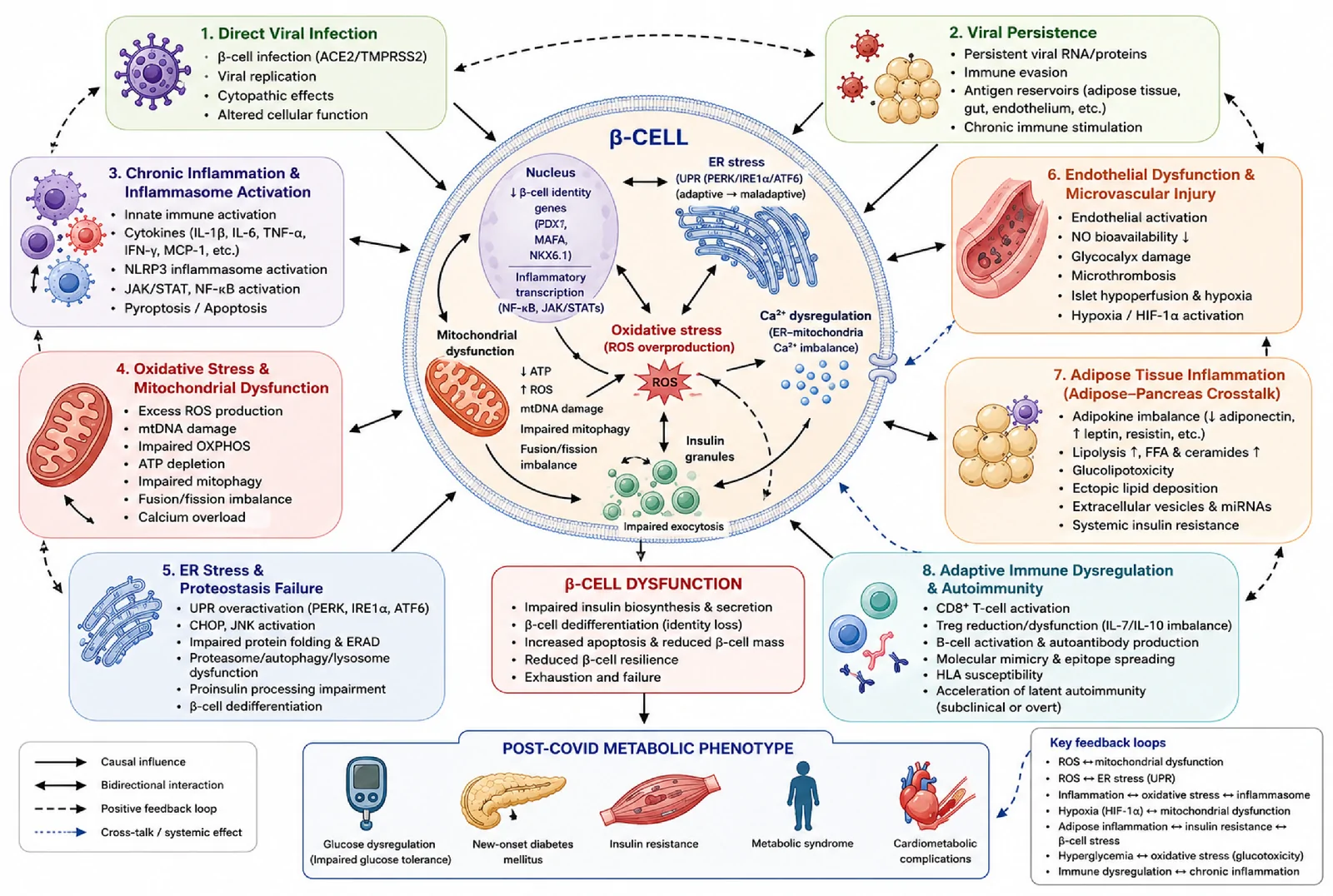
**Integrated mechanistic model of SARS-CoV-2-induced pancreatic β-cell dysfunction and post-COVID metabolic abnormalities.** SARS-CoV-2-associated β-cell dysfunction arises from the convergence of direct viral effects and interconnected pathogenic mechanisms, including viral persistence, chronic inflammation, oxidative stress, mitochondrial and endoplasmic reticulum dysfunction, endothelial injury, adipose tissue inflammation, and adaptive immune dysregulation. These processes interact through multiple positive feedback loops that impair insulin biosynthesis and secretion, promote β-cell dedifferentiation and apoptosis, and progressively reduce β-cell resilience. The cumulative disruption of pancreatic and systemic immunometabolic homeostasis contributes to the heterogeneous spectrum of post-COVID metabolic abnormalities, including impaired glucose regulation, insulin resistance, new-onset diabetes mellitus, metabolic syndrome, and cardiometabolic complications.

Generative artificial intelligence (OpenAI. ChatGPT (GPT-5.5). OpenAI, San Francisco, CA, USA) was used solely to assist in the graphical design of Figure 1. The authors were fully responsible for the literature search, critical evaluation of the evidence, scientific interpretation, manuscript writing, and final revision of the manuscript.

## 3. Physiological Basis of β-Cell Function

### 3.1. Glucose Sensing and Stimulus–Secretion Coupling

Accurate glucose sensing enables pancreatic β-cells to adjust insulin secretion in response to fluctuations in circulating glucose concentrations, thereby maintaining systemic glucose homeostasis. This highly coordinated process integrates glucose uptake, intracellular metabolism, mitochondrial ATP production, membrane excitability, calcium signalling, and insulin granule exocytosis. Disruption of any component of this pathway may impair insulin release and represents an early manifestation of β-cell dysfunction. Understanding these physiological mechanisms is therefore essential for interpreting how SARS-CoV-2-associated cellular stress may disturb β-cell function and contribute to post-COVID metabolic abnormalities [19,20].

Glucose enters pancreatic β-cells through facilitative glucose transporters, predominantly glucose transporter 1 (GLUT1) in humans and GLUT2 in rodents. This species-specific distinction is particularly important when extrapolating findings from experimental models to human β-cell biology. Regardless of the transporter involved, intracellular glucose metabolism provides the primary signal that initiates insulin secretion [19,20].

Following glucose uptake, glucokinase (GCK) functions as the principal metabolic glucose sensor by phosphorylating glucose and initiating glycolysis. Subsequent mitochondrial oxidative phosphorylation increases the intracellular ATP/ADP ratio, resulting in closure of ATP-sensitive potassium (K_ATP) channels, membrane depolarisation, calcium influx through voltage-dependent calcium channels, and calcium-triggered exocytosis of insulin-containing granules. Mitochondrial ATP production therefore constitutes the essential metabolic link between glucose sensing and insulin secretion [19,20].

Stimulus–secretion coupling relies on the close integration of glucose metabolism, mitochondrial bioenergetics, ion-channel activity, intracellular calcium dynamics, and vesicle exocytosis. Because these processes are functionally interdependent, impairment of one component can propagate throughout the secretory pathway. This physiological organisation helps explain how viral infection, chronic inflammation, oxidative stress, mitochondrial dysfunction, and endoplasmic reticulum stress may progressively compromise β-cell function in post-COVID metabolic disease.

### 3.2. Mitochondrial Homeostasis in β-Cell Function

Mitochondria represent the central metabolic hub linking glucose metabolism to insulin secretion. In addition to ATP generation, they regulate metabolic amplification, intracellular calcium handling, redox balance, and glucose-stimulated insulin secretion. Mitochondrial integrity is therefore indispensable for preserving β-cell secretory competence and systemic glucose homeostasis [19,21].

Mitochondrial function is sustained through continuous remodelling of the mitochondrial network. Fusion, mediated by mitofusin 1 (MFN1), mitofusin 2 (MFN2), and optic atrophy protein 1 (OPA1), preserves mitochondrial connectivity, respiratory efficiency, and metabolic competence. Conversely, fission, primarily regulated by dynamin-related protein 1 (DRP1), facilitates metabolic adaptation and the selective segregation of damaged mitochondrial components. Normal β-cell function depends not on the predominance of either process, but on a carefully regulated balance between fusion and fission [21,22,23].

Experimental deletion of OPA1 disrupts mitochondrial architecture and impairs glucose-stimulated ATP production, whereas loss of DRP1 alters mitochondrial organisation and reduces insulin secretion. These observations demonstrate that mitochondrial dynamics influence β-cell function through both bioenergetic and signalling mechanisms and are integral to normal stimulus–secretion coupling [22,23].

Mitochondrial competence also depends on coordinated biogenesis and quality-control pathways. Peroxisome proliferator-activated receptor-γ coactivator-1α (PGC-1α) contributes to the regulation of mitochondrial metabolic capacity. However, both insufficient and excessive PGC-1α activity may impair nutrient-coupled insulin secretion, indicating that balanced mitochondrial regulation is more important than maximal biogenesis [24,25].

Selective removal of dysfunctional mitochondria through mitophagy constitutes another essential component of β-cell quality control. Although the canonical PTEN-induced kinase 1 (PINK1)–Parkin pathway contributes to mitochondrial turnover, receptor-mediated and PINK1-independent mechanisms also participate in maintaining organelle integrity. Transcription factor EB (TFEB)-dependent activation of lysosomal pathways further supports mitochondrial respiration, limits oxidative injury, and preserves insulin secretory capacity during metabolic stress [26,27].

Reactive oxygen species (ROS) generated during mitochondrial respiration exert context-dependent effects on β-cell physiology. Low ROS concentrations may participate in metabolic signalling, whereas persistent ROS accumulation damages mitochondrial proteins, membrane lipids, and mitochondrial DNA, impairs ATP synthesis, and promotes β-cell dysfunction. Because pancreatic β-cells possess relatively limited antioxidant capacity, sustained redox regulation is particularly important for preserving mitochondrial and secretory function [14,15].

Mitochondrial bioenergetics, fusion–fission dynamics, biogenesis, mitophagy, calcium handling, and redox control thus form an integrated quality-control network. Disruption of this network compromises stimulus–secretion coupling and increases β-cell susceptibility to inflammatory, oxidative, hypoxic, metabolic, and viral stress, offering a mechanistic basis for the development of post-COVID metabolic dysfunction.

### 3.3. Calcium Signalling and Exocytotic Machinery in Pancreatic β-Cells

Intracellular calcium signalling represents the final common pathway coupling glucose metabolism to insulin secretion. Membrane depolarisation activates voltage-dependent calcium channels (VDCCs), resulting in rapid Ca^2+^ influx into pancreatic β-cells and initiation of insulin granule exocytosis. Precise control of intracellular calcium concentrations is therefore essential for normal β-cell function and systemic glucose homeostasis [19,20].

Rather than acting solely as an exocytotic trigger, intracellular Ca^2+^ displays coordinated oscillatory behaviour that determines both the magnitude and pulsatility of insulin secretion. These oscillations arise from the integrated activity of plasma membrane ion channels, mitochondrial ATP production, endoplasmic reticulum calcium stores, and intracellular buffering mechanisms. Their synchronisation across the islet enables β-cells to function as a coordinated multicellular network, including the insulin-mediated regulation of α-cell glucagon secretion. Effective stimulus–secretion coupling therefore depends not only on calcium influx but also on the spatial and temporal organisation of intracellular calcium dynamics [19,20,28,29].

The endoplasmic reticulum (ER) serves as the principal intracellular calcium reservoir and modulates cytosolic Ca^2+^ oscillations through coordinated calcium uptake by sarco/endoplasmic reticulum Ca^2+^-ATPase (SERCA) pumps and calcium release through inositol 1,4,5-trisphosphate receptors and ryanodine receptors. Functional communication between the ER and mitochondria at mitochondria-associated membranes (MAMs) further coordinates calcium transfer with mitochondrial metabolism and ATP production. Disturbance of this bidirectional communication may therefore rapidly impair β-cell secretory function [29,30,31].

The rise in cytosolic Ca^2+^ ultimately promotes fusion of insulin-containing granules with the plasma membrane through the soluble N-ethylmaleimide-sensitive factor attachment protein receptor (SNARE) machinery. This complex includes syntaxin-1A, synaptosome-associated protein 25 (SNAP-25), vesicle-associated membrane protein 2 (VAMP2), synaptotagmins, Munc18, and additional regulatory proteins. Normal insulin secretion consequently depends on both intact calcium signalling and the structural and functional integrity of the granule-docking and exocytotic apparatus [32,33].

Calcium signalling therefore represents a point of convergence between glucose metabolism, mitochondrial bioenergetics, ER function, and vesicle exocytosis. Disturbances in calcium homeostasis can propagate throughout the stimulus–secretion coupling pathway and impair insulin release even in morphologically preserved β-cells. This interdependence offers a physiological context for understanding how inflammation, oxidative stress, mitochondrial dysfunction, ER stress, and SARS-CoV-2-associated cellular injury may disrupt β-cell function.

### 3.4. Endoplasmic Reticulum Homeostasis and the Unfolded Protein Response in Pancreatic β-Cells

The endoplasmic reticulum is essential for pancreatic β-cell function because of its central roles in insulin biosynthesis, protein folding, post-translational processing, calcium storage, and intracellular protein quality control. Owing to their exceptionally high secretory activity, β-cells continuously synthesise substantial amounts of proinsulin, placing considerable demands on the ER folding machinery. Preservation of ER homeostasis is therefore critical for both β-cell viability and insulin secretory capacity [34,35].

Newly synthesised proinsulin enters the ER, where molecular chaperones, protein disulfide isomerases, and calcium-dependent folding mechanisms facilitate its correct maturation. Properly folded proinsulin is subsequently transported to the Golgi apparatus for further processing, whereas irreversibly misfolded proteins are removed through ER-associated degradation (ERAD), thereby preserving cellular proteostasis [34,35,36,37].

Under basal conditions, the ER chaperone binding immunoglobulin protein, also known as glucose-regulated protein 78 (BiP/GRP78), maintains the principal ER stress sensors in an inactive state. Accumulation of unfolded or misfolded proteins sequesters BiP and activates the unfolded protein response (UPR) through protein kinase RNA-like ER kinase (PERK), inositol-requiring enzyme 1α (IRE1α), and activating transcription factor 6 (ATF6). Adaptive UPR signalling transiently reduces global protein translation, increases chaperone expression, and promotes the clearance of misfolded proteins, thereby supporting restoration of ER homeostasis [35,36,37].

When ER stress becomes prolonged or excessive, however, the initially protective response may shift towards maladaptive and pro-apoptotic signalling. This transition involves CCAAT/enhancer-binding protein homologous protein (CHOP), c-Jun N-terminal kinase activation, oxidative stress, mitochondrial dysfunction, and ultimately β-cell apoptosis [35,36,37,38].

The ER is closely connected to mitochondria through MAMs, which coordinate calcium exchange, ATP generation, lipid metabolism, and cellular stress responses. Physiological ER–mitochondrial communication supports insulin biosynthesis and secretion, whereas disruption of these contact sites may promote mitochondrial calcium overload, oxidative injury, and β-cell dysfunction. These interactions highlight the close functional interdependence of ER proteostasis, mitochondrial integrity, and intracellular calcium signalling [30,31].

ER protein quality control, adaptive UPR signalling, and ER–mitochondrial communication therefore constitute essential components of β-cell homeostasis. Failure of this coordinated system compromises insulin biosynthesis, secretory function, and cell survival, helping to explain the particular vulnerability of pancreatic β-cells to inflammatory, metabolic, oxidative, and viral stress.

### 3.5. β-Cell Plasticity, Functional Heterogeneity, and Adaptive Responses

Pancreatic β-cells were once considered a relatively homogeneous population of insulin-producing cells. Advances in single-cell transcriptomics, spatial biology, electrophysiology, and high-resolution imaging have fundamentally changed this view, demonstrating that pancreatic islets contain multiple β-cell subpopulations with distinct transcriptional, metabolic, electrophysiological, and secretory characteristics. Rather than functioning as identical units, β-cells form a dynamic and heterogeneous cellular network capable of adapting to changing metabolic demands [39,40,41,42,43].

This heterogeneity contributes to coordinated insulin secretion while preserving metabolic flexibility. Within the islet network, highly connected hub or leader β-cells can coordinate electrical activity and synchronise calcium oscillations, enabling the islet to function as an integrated multicellular system rather than as a collection of independent secretory cells [40,44].

β-cells also possess substantial functional plasticity. During physiological adaptation, including pregnancy, and in states of increased insulin demand, such as obesity and insulin resistance, β-cells may enhance insulin biosynthesis and secretory activity to preserve glucose homeostasis. When metabolic stress persists, however, compensatory capacity may progressively decline, resulting in impaired insulin secretion, loss of β-cell identity, and functional failure [45,46].

A prominent response to sustained cellular stress is β-cell dedifferentiation, during which mature β-cells lose key features of their differentiated phenotype and acquire a less specialised state. This process may initially reduce the metabolic and secretory burden placed on the cell. Nevertheless, prolonged dedifferentiation can limit redifferentiation, diminish functional β-cell mass, and contribute to chronic insulin deficiency [45,46].

β-cell heterogeneity and plasticity are therefore important determinants of metabolic resilience. Interindividual differences in functional reserve, cellular composition, and adaptive capacity may partly explain why some patients recover normal glucose regulation after SARS-CoV-2 infection, whereas others develop persistent dysglycaemia or newly diagnosed diabetes. These principles provide a physiological basis for the heterogeneous clinical manifestations of post-COVID metabolic dysfunction [39,40,41,42,43,44,45,46].

### 3.6. Integration of Physiological Mechanisms Underlying β-Cell Function

Physiological insulin secretion depends on the coordinated interaction of multiple intracellular processes rather than on isolated molecular pathways. Glucose metabolism, mitochondrial bioenergetics, ion-channel activity, calcium signalling, ER proteostasis, vesicle trafficking, and β-cell plasticity operate as an integrated regulatory network that preserves insulin biosynthesis, stimulus–secretion coupling, and metabolic homeostasis [19,20,34].

This interconnected organisation provides substantial metabolic flexibility but also creates multiple points of vulnerability. Disturbances affecting an individual pathway may initially be offset by adaptive responses, including mitochondrial quality control, UPR activation, enhanced secretory capacity, and β-cell plasticity. However, the simultaneous or sustained disruption of several interdependent mechanisms progressively exceeds compensatory capacity and promotes secretory failure [30,32,40,46].

β-cell dysfunction should therefore be viewed as the consequence of failure of an integrated biological network rather than as an isolated defect in a single molecular pathway. This systems-level perspective helps explain how inflammatory, oxidative, metabolic, hypoxic, and viral insults converge to impair β-cell function and generate the heterogeneous metabolic manifestations observed following SARS-CoV-2 infection.

## 4. Mechanisms of β-Cell Dysfunction in COVID-19 and Post-COVID Conditions

Pancreatic β-cell dysfunction associated with SARS-CoV-2 infection arises from the convergence of direct viral effects and multiple secondary pathogenic processes. These include persistent viral antigens, chronic inflammation, dysregulated cytokine signalling, oxidative stress, mitochondrial and endoplasmic reticulum dysfunction, endothelial injury, altered intracellular calcium homeostasis, and impaired immune resolution. Rather than operating independently, these mechanisms form an interconnected network capable of disrupting insulin biosynthesis, stimulus–secretion coupling, cellular stress adaptation, and β-cell survival.

The relative contribution of each pathway is likely to differ according to viral burden, disease severity, host genetic susceptibility, pre-existing metabolic status, age, obesity, and the efficiency of antiviral and immunoregulatory responses. This biological heterogeneity may partly explain why glucose abnormalities resolve in some individuals following acute infection, whereas others develop persistent dysglycaemia, progressive β-cell dysfunction, or newly diagnosed diabetes. The following subsections examine the principal mechanisms through which SARS-CoV-2 may impair pancreatic β-cell function during acute COVID-19 and throughout the post-acute period.

### 4.1. Direct Viral Infection of Pancreatic β-Cells: Receptor Expression and Mechanisms of Viral Entry

Direct infection of pancreatic β-cells has been proposed as one mechanism contributing to COVID-19-associated metabolic dysfunction. Nevertheless, whether SARS-CoV-2 efficiently and consistently infects human β-cells in vivo remains one of the most debated questions in the field. Available experimental and pathological evidence suggests that β-cell injury is unlikely to result exclusively from direct viral cytotoxicity. Instead, viral entry may cooperate with inflammatory signalling, metabolic stress, endothelial dysfunction, and immune-mediated injury. Distinguishing direct infection from secondary mechanisms is therefore essential for interpreting the pathogenesis of post-COVID metabolic disease [47,48,49].

SARS-CoV-2 initiates cellular entry through binding of its spike glycoprotein to angiotensin-converting enzyme 2 (ACE2). Successful entry also requires proteolytic processing of the spike protein. Transmembrane serine protease 2 (TMPRSS2) promotes fusion at the plasma membrane, whereas furin-mediated cleavage may pre-activate the spike protein before cellular entry. Cathepsins B and L support an alternative endosomal route, particularly in cells with limited TMPRSS2 expression. Other proteases, including TMPRSS4, may complement these pathways, while a disintegrin and metalloproteinase 17 (ADAM17) can modulate ACE2 shedding and thereby influence receptor availability and local inflammatory signalling. Viral tropism is consequently determined not by receptor expression alone, but by the combined availability of entry receptors, cofactors, and proteolytic enzymes within the target tissue [50,51,52,53,54].

Several additional host factors have been investigated as potential facilitators of SARS-CoV-2 entry. Neuropilin-1 (NRP1) can enhance infectivity after furin-mediated spike cleavage and may broaden cellular susceptibility when ACE2 expression is relatively low. CD147, also known as basigin, and dipeptidyl peptidase-4 (DPP4) have likewise been proposed as accessory receptors or entry cofactors. Evidence supporting their involvement in productive SARS-CoV-2 infection, however, remains inconsistent, and their specific relevance to pancreatic β-cells has not been established. β-cell susceptibility should therefore be interpreted as the product of a complex local entry environment rather than as a direct consequence of ACE2 expression alone [52,53,54,55].

Substantial controversy persists regarding the abundance and cellular localisation of SARS-CoV-2 entry factors within the human pancreas (Table 1). Some transcriptomic and immunohistochemical studies have detected ACE2 in endocrine cells and the pancreatic microvasculature, whereas others have reported that ACE2 and TMPRSS2 are primarily enriched in ducts, endothelial cells, and perivascular compartments rather than in β-cells [48,49,56]. These discrepancies may reflect differences in tissue preservation, antibody specificity, analytical sensitivity, disease stage, and cellular annotation. Importantly, the detection of ACE2 messenger RNA does not necessarily indicate abundant functional protein at the cell surface, and protein immunoreactivity does not by itself demonstrate receptor accessibility or productive infection. Species-specific differences between human and rodent pancreatic tissue further complicate the extrapolation of experimental findings. Receptor expression should therefore be regarded as evidence of biological plausibility rather than definitive proof of viral tropism.

Studies using isolated human islets and experimental pancreatic models indicate that SARS-CoV-2 can infect endocrine pancreatic cells under selected conditions. Reported consequences include impaired insulin secretion, mitochondrial injury, activation of cellular stress pathways, altered β-cell identity, and apoptosis [47,49]. Nevertheless, the extent to which these findings reproduce the in vivo microenvironment remains uncertain. Analyses of pancreatic tissue from individuals with COVID-19 have also produced heterogeneous results, with viral RNA or proteins detected in some samples but absent or confined to non-endocrine compartments in others [47,48,49,56]. Direct β-cell infection may therefore occur in selected individuals or clinical contexts without representing a universal mechanism of post-COVID metabolic dysfunction.

Overall, the available evidence supports the biological possibility of direct SARS-CoV-2 infection of pancreatic β-cells but does not establish it as the sole or predominant cause of post-COVID dysglycaemia. β-cell susceptibility appears to depend on the interaction among receptor abundance, protease activity, viral tropism, tissue vascularisation, inflammatory signalling, and the local pancreatic microenvironment. Direct viral effects should consequently be interpreted within the broader network of secondary inflammatory, vascular, mitochondrial, and metabolic mechanisms described below.

### 4.2. Viral Persistence and Chronic Cellular Stress

Although COVID-19 is initiated as an acute viral infection, complete elimination of viral material may not occur in every individual. Experimental, pathological, and clinical studies have detected SARS-CoV-2 RNA, viral proteins, and, less commonly, replication-competent virus in extrapulmonary tissues after resolution of the initial illness. Viral persistence has consequently emerged as one of several leading hypotheses for the pathogenesis of Long COVID and may contribute to sustained immune activation, chronic inflammation, and prolonged metabolic dysfunction [57,58,59].

Viral persistence should not be equated with continuous productive replication in all affected individuals. Persistent material may consist of residual RNA fragments, intracellular proteins, replication-defective particles, or infectious virus, each of which may have different biological consequences. Viral RNA or antigen detection alone therefore cannot establish ongoing replication. Interpretation requires consideration of the tissue compartment examined, the detection method used, the integrity of the viral material, and evidence of active transcription or viral culture [57,58,59].

Autopsy and tissue-based studies have identified persistent viral components in several extrapulmonary organs. The gastrointestinal tract is among the most consistently proposed reservoirs, while adipose tissue, lymphoid tissue, and tissue-resident immune cells may also retain viral antigens. Viral RNA or proteins have been reported in pancreatic tissue in selected investigations, although the frequency, duration, cellular localisation, and clinical significance of pancreatic persistence remain uncertain [57,58,59,60,61]. Tissue reservoirs outside the pancreas may nevertheless influence β-cell function indirectly by maintaining systemic antigenic exposure and a prolonged inflammatory environment.

Several mechanisms could permit the prolonged retention of viral material. These include incomplete elimination of infected cells, impaired type I interferon responses, viral immune evasion, infection of long-lived tissue-resident cells, macrophage-associated antigen retention, and reduced antiviral immune surveillance. Such processes need not act independently. Their combined effects may sustain low-level antigenic stimulation and prevent complete restoration of innate and adaptive immune homeostasis after apparent clinical recovery [57,60,62,63,64].

Persistent viral antigens may influence glucose regulation by acting as amplifiers of several interconnected stress pathways. Continued antigenic stimulation can maintain cytokine production, activate innate immune sensors and the NLR family pyrin domain-containing 3 (NLRP3) inflammasome, increase oxidative stress, disturb mitochondrial and endoplasmic reticulum homeostasis, impair endothelial function, and alter intracellular calcium signalling. Through these mechanisms, persistent viral material could progressively exceed β-cell adaptive capacity and impair insulin secretion, even without continuous viral replication within β-cells themselves [60,61,62,63,64].

The existing evidence nevertheless remains heterogeneous. Studies differ substantially in the populations examined, tissues sampled, timing of assessment, detection technologies, and criteria used to define persistence. Furthermore, viral reservoirs are unlikely to be present or clinically relevant in all individuals with Long COVID or post-COVID metabolic abnormalities. Their effects may be confined to susceptible subgroups and modified by host genetics, immune competence, viral burden, baseline metabolic health, and pre-existing comorbidities.

Viral persistence should therefore be regarded as a plausible but non-universal contributor to post-COVID metabolic dysfunction. In affected individuals, residual viral antigens or tissue reservoirs may maintain inflammatory and metabolic stress and thereby connect the acute infection with the chronic immune, mitochondrial, endothelial, and β-cell abnormalities that emerge during the post-acute period.

### 4.3. Chronic Inflammation, Cytokine Signalling, and Inflammasome Activation in β-Cell Dysfunction

Persistent immune activation represents a major biological link between acute SARS-CoV-2 infection and long-term metabolic dysfunction. Although the intense inflammatory response associated with severe COVID-19 usually declines after the acute phase, low-grade inflammation and altered immune signalling may persist for months. Sustained cytokine production, incomplete immune reconstitution, and dysregulated immunometabolic responses can collectively impair pancreatic β-cell function and glucose homeostasis [5,58,62,64].

Among the inflammatory mediators implicated in β-cell injury, interleukin-1β (IL-1β), interleukin-6 (IL-6), tumour necrosis factor-α (TNF-α), interferon-γ (IFN-γ), and type I interferons are particularly relevant. These cytokines converge on intracellular pathways that include nuclear factor kappa B (NF-κB), Janus kinase/signal transducer and activator of transcription (JAK/STAT), mitogen-activated protein kinase (MAPK), and c-Jun N-terminal kinase (JNK). Persistent activation of these pathways can suppress insulin gene expression, impair glucose-stimulated insulin secretion, promote oxidative and endoplasmic reticulum stress, and activate pro-apoptotic programmes [65,66,67].

IL-1β is a well-established mediator of inflammatory β-cell injury. Prolonged exposure activates NF-κB-dependent transcription, disrupts mitochondrial ATP production, and can impair insulin biosynthesis and secretion. TNF-α reinforces these effects by promoting oxidative stress, mitochondrial dysfunction, inflammatory signalling, and peripheral insulin resistance. The actions of IL-6 are more context-dependent: transient signalling may contribute to tissue adaptation and host defence, whereas sustained elevation can support chronic inflammation, altered insulin action, and progressive metabolic dysfunction [65,66,67].

Post-COVID immune dysregulation may also reflect defective resolution of inflammation rather than persistent pro-inflammatory activation alone. Restoration of immune homeostasis requires effective immunoregulatory signalling and reconstitution of lymphocyte populations. In this context, the IL-7/IL-10 axis is of particular interest. IL-7 supports T-cell survival, homeostatic proliferation, and immune recovery, whereas IL-10 restrains excessive innate and adaptive immune activation. Disturbances in these pathways could permit inflammatory signalling to persist despite apparent clinical resolution of the acute infection.

Immunometabolic profiling has identified alterations in circulating IL-7 and IL-10 concentrations among individuals who developed metabolic abnormalities after SARS-CoV-2 infection [11]. These observational findings do not demonstrate a direct causal effect on pancreatic β-cells. Nevertheless, they support the possibility that incomplete immune resolution contributes indirectly to β-cell dysfunction by maintaining inflammatory signalling, oxidative stress, mitochondrial impairment, and systemic metabolic imbalance.

Activation of the NLRP3 inflammasome provides an additional mechanistic link between cellular stress and inflammation. NLRP3 integrates signals generated by mitochondrial dysfunction, reactive oxygen species, ionic disturbances, and metabolic stress. Its activation promotes caspase-1-dependent maturation of IL-1β and interleukin-18 (IL-18), thereby reinforcing inflammatory responses and establishing reciprocal feedback between mitochondrial injury, oxidative stress, and cytokine production [67,68]. Persistent inflammasome activity may interfere with insulin secretion, calcium homeostasis, and β-cell survival, although direct evidence in human pancreatic tissue after COVID-19 remains limited.

Chronic inflammation is also accompanied by immunometabolic reprogramming. Activated immune cells frequently increase glycolytic flux, alter mitochondrial substrate utilisation, and modify biosynthetic pathways to sustain effector functions and cytokine production. Although these adaptations are essential during acute host defence, their persistence can support chronic inflammatory phenotypes and influence pancreatic β-cells through systemic and paracrine mediators [69,70]. Inflammation should therefore be understood not only as an immune response but also as a metabolic process capable of modifying whole-body glucose regulation.

These mechanisms rarely operate independently. Persistent cytokine exposure interacts with viral antigens, mitochondrial dysfunction, oxidative stress, endoplasmic reticulum stress, endothelial injury, and defective immunoregulation. The resulting feedback network may progressively reduce β-cell adaptive capacity and contribute to the biological heterogeneity of post-COVID metabolic dysfunction. Longitudinal immunophenotyping, single-cell analyses, and studies integrating immune and metabolic biomarkers will be required to identify distinct inflammatory endotypes and establish their clinical relevance.

Overall, chronic inflammation represents a central but heterogeneous mechanism of post-COVID β-cell dysfunction. Its effects arise through the combined actions of pro-inflammatory cytokines, impaired immune resolution, inflammasome activation, and metabolic reprogramming rather than through a single inflammatory mediator.

### 4.4. Oxidative Stress, Mitochondrial Dysfunction, and Redox Imbalance in SARS-CoV-2-Induced β-Cell Injury

Oxidative stress and mitochondrial dysfunction form a closely interconnected mechanism linking SARS-CoV-2 infection with persistent β-cell impairment. Mitochondrial injury promotes excessive production of reactive oxygen species (ROS), while sustained ROS accumulation further damages mitochondrial proteins, lipids, and nucleic acids. This reciprocal interaction can progressively impair bioenergetics, insulin secretion, and β-cell viability, thereby connecting chronic inflammation with post-COVID metabolic dysfunction [19,71,72].

Under physiological conditions, mitochondria generate ATP required for glucose-stimulated insulin secretion and produce low concentrations of ROS that participate in metabolic signalling. Pancreatic β-cells, however, possess relatively limited antioxidant capacity compared with many other tissues. Persistent ROS accumulation can therefore shift physiological redox signalling towards oxidative injury, leading to lipid peroxidation, protein modification, mitochondrial DNA damage, and impaired oxidative phosphorylation [71,72].

Several processes may increase ROS production during and after SARS-CoV-2 infection. Pro-inflammatory cytokines can activate nicotinamide adenine dinucleotide phosphate (NADPH) oxidases, while virus-associated mitochondrial perturbation may increase electron leakage from the respiratory chain. Mitochondrial ROS can subsequently activate NF-κB and the NLRP3 inflammasome, which further increase cytokine production and oxidative stress. In this manner, inflammation and redox imbalance form a self-reinforcing cycle rather than a unidirectional sequence of events [23,67,72,73].

Mitochondria also undergo continuous structural remodelling through fusion and fission. These processes allow β-cells to adapt mitochondrial architecture to changing metabolic demands and to segregate damaged organelles for removal. SARS-CoV-2 has been proposed to alter host mitochondrial metabolism, antiviral signalling, and organelle quality-control pathways [73]. Independently, experimental β-cell studies demonstrate that disruption of mitochondrial dynamics impairs ATP production, metabolic amplification, calcium handling, and insulin secretion [21,23]. Defective mitophagy may further permit damaged mitochondria to accumulate, reducing bioenergetic efficiency and amplifying ROS generation.

Mitochondrial dysfunction can also disturb intracellular calcium homeostasis. Reduced ATP availability impairs energy-dependent ion transport, while damaged mitochondria exhibit diminished capacity to buffer cytosolic calcium. Excessive or prolonged mitochondrial calcium loading may promote permeability transition, loss of membrane potential, and activation of intrinsic apoptotic pathways. At the functional level, these abnormalities can impair both the triggering and amplifying phases of glucose-stimulated insulin secretion [19,23,71].

Viral infection and antiviral immune activation may additionally induce metabolic reprogramming characterised by enhanced glycolytic activity and reduced mitochondrial respiration [21,23,74]. Such adaptation can support rapid immune and cellular defence during acute infection. Prolonged suppression of oxidative phosphorylation, however, would be particularly detrimental to β-cells because glucose-stimulated insulin secretion depends strongly on mitochondrial ATP generation. Direct evidence demonstrating persistent glycolytic reprogramming specifically in human pancreatic β-cells after SARS-CoV-2 infection remains limited, and this mechanism should therefore be regarded as biologically plausible rather than definitively established.

Oxidative stress is not merely a downstream consequence of impaired mitochondrial function. Excessive ROS can aggravate endoplasmic reticulum stress, activate inflammasome pathways, modify calcium-handling proteins, impair endothelial function, and disrupt insulin synthesis and granule exocytosis [75]. Conversely, damaged mitochondria release mitochondrial DNA, oxidised lipids, metabolites, and other danger-associated signals that amplify innate immune responses. These reciprocal interactions convert mitochondrial dysfunction, redox imbalance, and inflammation into an integrated pathogenic network [23,72,74,75,76].

Interpretation of the available evidence requires caution. Many mechanistic observations originate from cultured cells, animal models, or stem-cell-derived β-like cells. Direct longitudinal evidence of persistent mitochondrial dysfunction within human pancreatic β-cells after COVID-19 remains scarce. The extent of mitochondrial injury is also likely to be influenced by age, adiposity, pre-existing diabetes, genetic susceptibility, disease severity, viral burden, medication exposure, and baseline metabolic health.

Taken together, mitochondrial dysfunction represents an integrative hub connecting oxidative stress, inflammatory signalling, impaired calcium homeostasis, defective organelle quality control, and progressive β-cell failure. These pathways reinforce one another and may contribute substantially to post-COVID metabolic abnormalities (Table 2). Their persistence and clinical significance in human pancreatic tissue, however, require confirmation through longitudinal studies combining functional mitochondrial assays, metabolomics, tissue imaging, and spatially resolved molecular analyses.

### 4.5. SARS-CoV-2-Induced Endoplasmic Reticulum Stress and Proteostasis Failure

The endoplasmic reticulum (ER) is central to pancreatic β-cell function because it coordinates insulin biosynthesis, protein folding, calcium storage, and intracellular protein quality control. Owing to their continuously high secretory demand, β-cells are particularly dependent on efficient ER proteostasis and are highly susceptible to disturbances in protein-folding capacity. During SARS-CoV-2 infection, viral protein synthesis, inflammatory signalling, oxidative stress, and mitochondrial dysfunction may collectively increase the ER burden, progressively exceeding adaptive capacity and compromising β-cell function [34,36,37].

Coronaviruses extensively exploit ER-derived membranes for viral protein synthesis, processing, and formation of replication organelles. SARS-CoV-2 replication therefore places additional demands on the host secretory pathway and can alter ER membrane architecture and function. Although these processes are essential for viral replication, their consequences extend beyond infected cells by activating cellular stress responses and amplifying inflammatory and metabolic dysfunction [77,78,79,80].

ER stress activates the unfolded protein response (UPR), which is mediated through the inositol-requiring enzyme 1α (IRE1α), protein kinase RNA-like ER kinase (PERK), and activating transcription factor 6 (ATF6) pathways. During transient stress, the UPR promotes adaptation by attenuating global protein translation, increasing molecular chaperone expression, and enhancing the degradation of misfolded proteins. Persistent or excessive activation, however, can shift the response from restoration of proteostasis towards maladaptive signalling involving C/EBP homologous protein (CHOP), c-Jun N-terminal kinase (JNK), and other pro-apoptotic pathways. In β-cells, this transition may impair proinsulin synthesis and processing, suppress insulin secretion, and reduce cellular viability [34,36,37,81].

ER homeostasis is closely connected to mitochondrial function through specialised contact sites that coordinate lipid exchange, calcium transfer, and metabolic signalling. Excessive mitochondrial ROS can increase oxidative protein damage within the ER, whereas uncontrolled ER calcium release can promote mitochondrial calcium overload, impair oxidative phosphorylation, and reduce ATP production. These reciprocal interactions establish a self-reinforcing cycle in which ER and mitochondrial stress progressively reduce β-cell functional reserve [30,31].

SARS-CoV-2-associated cellular stress may also interfere with protein quality-control systems beyond the canonical UPR. These include ER-associated degradation, the ubiquitin–proteasome system, autophagy, and lysosomal clearance. Impairment of these pathways permits the accumulation of misfolded proteins and dysfunctional organelles, further intensifying proteotoxic and oxidative stress [34,36,37,78,79,80,81]. In β-cells, deterioration of protein quality control may disrupt proinsulin folding and conversion before overt cell death becomes evident. Proteostasis failure may therefore represent an early functional abnormality rather than merely a terminal consequence of irreversible β-cell injury.

Sustained ER stress may additionally contribute to loss of mature β-cell identity. Experimental models of metabolic stress have linked prolonged activation of stress-response pathways with reduced expression of transcription factors required for differentiated β-cell function, including pancreatic and duodenal homeobox 1 (PDX1), musculoaponeurotic fibrosarcoma oncogene family A (MAFA), and NK6 homeobox 1 (NKX6.1). The resulting reduction in insulin gene transcription and secretory competence suggests that β-cell failure may involve potentially reversible dedifferentiation as well as apoptosis and loss of functional cell mass [45,65].

ER stress should therefore not be considered an isolated mechanism. It integrates signals generated by viral replication or persistence, inflammatory cytokines, oxidative stress, mitochondrial dysfunction, altered calcium homeostasis, endothelial injury, and impaired autophagic clearance. The cumulative burden of these disturbances may progressively shift β-cells from adaptive UPR activation towards persistent secretory dysfunction, dedifferentiation, and cell death.

Most evidence linking SARS-CoV-2 to sustained ER dysfunction derives from experimental systems, studies of individual viral proteins, or broader coronavirus biology. Direct demonstration of persistent ER stress in human pancreatic β-cells after COVID-19 remains limited. It is also uncertain whether ER dysfunction is initiated primarily by viral processes within pancreatic cells or develops predominantly as a secondary consequence of chronic inflammation, oxidative injury, and altered metabolic demand.

Overall, ER stress represents an important integrative mechanism connecting disturbed proteostasis with mitochondrial dysfunction, oxidative stress, calcium dysregulation, altered β-cell identity, and impaired insulin biosynthesis. Further studies using human pancreatic tissue, single-cell transcriptomics, proteomics, and spatial molecular profiling will be necessary to establish its persistence and relative contribution to post-COVID metabolic disease.

### 4.6. Endothelial Dysfunction and Microvascular Injury in SARS-CoV-2-Induced β-Cell Dysfunction

In addition to direct viral and intracellular mechanisms, endothelial dysfunction may contribute substantially to COVID-19-associated metabolic abnormalities. SARS-CoV-2 infection and the accompanying systemic inflammatory response can produce endothelial activation, vascular barrier disruption, coagulation abnormalities, and impaired microvascular perfusion. Endothelial injury should therefore be regarded not merely as a systemic complication of COVID-19 but also as a potential determinant of the pancreatic islet microenvironment and β-cell function [82,83,84].

Human pancreatic islets are supported by a highly specialised microvascular network that enables the rapid delivery of oxygen, glucose, nutrients, and circulating hormones while facilitating the efficient release of insulin into the bloodstream [85,86,87]. Islet endothelial cells and pericytes also regulate capillary diameter, local blood flow, extracellular matrix composition, and paracrine communication with endocrine cells [86,87]. Consequently, disruption of vascular homeostasis may impair β-cell function even in the absence of direct infection of endocrine cells.

COVID-19-associated endothelial injury arises through several complementary mechanisms, including inflammatory cytokine signalling, oxidative stress, complement activation, platelet activation, coagulation disturbances, and, in selected vascular beds, possible direct viral effects. These processes reduce nitric oxide bioavailability, impair vasodilatory responses, increase endothelial permeability, and promote microvascular thrombosis, collectively limiting tissue oxygenation and nutrient delivery [82,83,84,88,89].

Damage to the endothelial glycocalyx may further intensify microvascular dysfunction. The glycocalyx is a specialised luminal surface layer involved in vascular barrier integrity, mechanotransduction, regulation of leukocyte adhesion, and maintenance of anticoagulant properties. Its degradation promotes vascular leakage, inflammatory-cell recruitment, platelet adhesion, and prothrombotic signalling [88]. Although direct evidence from pancreatic islets remains limited, systemic glycocalyx injury and microthrombotic vascular changes could plausibly compromise islet perfusion during and after SARS-CoV-2 infection [88,89].

Pancreatic β-cells have high metabolic and oxygen requirements because glucose-stimulated insulin secretion depends strongly on oxidative phosphorylation and ATP generation. Even moderate reductions in local blood flow may therefore impair stimulus–secretion coupling. Persistent microvascular dysfunction could also amplify mitochondrial injury, oxidative stress, endoplasmic reticulum stress, and inflammatory signalling, establishing an additional feedback loop between vascular and endocrine dysfunction [85,86,87].

Impaired capillary perfusion and microthrombus formation may contribute to local tissue hypoxia. Hypoxia-inducible factors, particularly hypoxia-inducible factor-1α (HIF-1α), initially coordinate adaptive responses by increasing glycolytic capacity and promoting cellular survival under reduced oxygen availability. Prolonged or excessive HIF signalling, however, can suppress mitochondrial oxidative metabolism, alter redox homeostasis, and interfere with cellular differentiation and secretory function [90,91,92]. In pancreatic β-cells, persistent hypoxia may consequently reduce ATP production and impair glucose-stimulated insulin secretion.

The vascular and metabolic effects of hypoxia are likely to depend on duration and severity. Acute HIF activation may be protective by facilitating cellular adaptation, whereas chronic hypoxic signalling may promote glycolytic reprogramming, mitochondrial dysfunction, and oxidative injury. This distinction is particularly relevant to COVID-19, in which severe systemic hypoxaemia during acute illness may be followed by more subtle microvascular abnormalities during recovery.

Endothelial dysfunction also interacts continuously with inflammatory cytokines, oxidative stress, viral antigens, mitochondrial injury, ER stress, and dysregulated immune responses. These interactions support a multicellular model in which β-cell dysfunction reflects disruption of the entire islet microenvironment rather than injury confined exclusively to endocrine cells.

Several uncertainties nevertheless remain. Direct longitudinal evidence demonstrating persistent microvascular injury within human pancreatic islets is scarce, and much of the available knowledge is extrapolated from systemic vascular studies, pulmonary pathology, or experimental models. It is also unclear whether endothelial dysfunction initiates pancreatic injury or primarily amplifies β-cell stress produced by systemic inflammation and metabolic imbalance.

Taken together, endothelial dysfunction represents a biologically plausible amplifier of SARS-CoV-2-associated β-cell injury. Impaired endothelial integrity, altered perfusion, microthrombosis, and disturbed oxygen delivery may converge with intracellular stress pathways to compromise insulin secretion and β-cell survival. High-resolution vascular imaging, spatial transcriptomics, and single-cell analyses of human pancreatic tissue will be needed to define the magnitude and persistence of these effects.

### 4.7. Adipose Tissue–Pancreas Crosstalk in Post-COVID Metabolic Dysfunction

Adipose tissue is an active endocrine and immunometabolic organ that plays a central role in systemic glucose homeostasis. In addition to storing energy, adipocytes and stromal immune cells release adipokines, cytokines, lipid mediators, metabolites, and extracellular vesicles that regulate insulin sensitivity, inflammatory responses, vascular function, and pancreatic β-cell adaptation [93,94,95,96]. Disturbance of adipose tissue homeostasis following SARS-CoV-2 infection may therefore contribute indirectly to persistent β-cell dysfunction.

Experimental and pathological findings indicate that SARS-CoV-2 can affect human adipose tissue, including adipocytes and adipose-resident macrophages. Viral exposure within this compartment is associated with inflammatory-cell activation and increased cytokine production [97,98]. Whether adipose tissue serves as a long-term reservoir of replication-competent virus remains uncertain, but persistent viral antigens or prolonged inflammatory remodelling within a large and metabolically active organ could exert systemic effects after apparent recovery from acute infection.

Inflammatory remodelling alters the adipose secretory profile. Reduced adiponectin and increased leptin, resistin, and inflammatory cytokines can promote systemic insulin resistance, endothelial dysfunction, oxidative stress, and chronic low-grade inflammation [93,94,95,96]. The resulting increase in insulin demand places additional pressure on pancreatic β-cells. In individuals with limited functional reserve, persistent insulin resistance may accelerate the transition from compensatory hyperinsulinaemia to inadequate insulin secretion and dysglycaemia.

Adipose tissue dysfunction also increases the release of non-esterified fatty acids, ceramides, and other bioactive lipid species. Chronic exposure of β-cells to excessive lipid substrates can induce mitochondrial dysfunction, ROS production, ER stress, altered calcium signalling, and activation of inflammatory and apoptotic pathways [99,100,101]. When lipid excess occurs together with hyperglycaemia, glucolipotoxicity may further impair insulin gene expression, proinsulin processing, and glucose-stimulated insulin secretion.

The metabolic consequences of adipose inflammation extend beyond the direct effects of circulating lipids and cytokines. Adipose-derived extracellular vesicles can carry microRNAs, proteins, and bioactive lipids to distant tissues and modify gene expression and metabolic function [102]. This form of inter-organ communication may influence pancreatic islets, although direct evidence linking specific adipose-derived extracellular vesicles to β-cell dysfunction after COVID-19 is currently insufficient. Their potential value as biomarkers or therapeutic targets remains an important area for future investigation.

Communication between adipose tissue and pancreatic β-cells is bidirectional. Insulin regulates adipose glucose uptake, lipid storage, and suppression of lipolysis. Progressive β-cell dysfunction and relative insulin deficiency can therefore increase adipose lipolysis and circulating lipid concentrations, further aggravating insulin resistance and lipotoxic β-cell stress. This reciprocal relationship establishes a feedback loop that may be particularly relevant in individuals with obesity, visceral adiposity, or metabolic syndrome [93,94,99,100,101].

Adipose tissue fibrosis and impaired angiogenesis may further contribute to metabolic dysfunction by promoting local hypoxia, macrophage recruitment, and altered adipokine production [103]. These processes connect adipose pathology with the endothelial, hypoxic, inflammatory, and mitochondrial mechanisms described in the preceding sections. The adipose tissue–pancreas axis should consequently be considered part of a broader inter-organ network rather than an isolated endocrine pathway.

The clinical significance of this axis in post-COVID disease remains incompletely defined. Most available mechanistic evidence originates from studies of obesity, metabolic syndrome, and type 2 diabetes, whereas direct longitudinal data linking post-COVID adipose abnormalities with dynamic measurements of β-cell function are limited. The relative importance of adipokines, lipid mediators, extracellular vesicles, local fibrosis, and possible viral persistence is also likely to vary among individuals.

Overall, adipose tissue dysfunction may amplify post-COVID metabolic abnormalities through insulin resistance, inflammatory signalling, altered adipokine secretion, lipotoxicity, and inter-organ communication. Persistent disruption of adipose tissue–pancreas crosstalk may therefore contribute to the heterogeneous metabolic phenotypes observed after SARS-CoV-2 infection, particularly among individuals with pre-existing adiposity or limited β-cell reserve.

### 4.8. Adaptive Immune Dysregulation, Autoimmunity, and β-Cell Injury

Although innate immune activation dominates the early response to SARS-CoV-2, abnormalities of adaptive immunity may persist after resolution of the acute infection. Longitudinal studies have described incomplete restoration of lymphocyte compartments, sustained immune activation, altered differentiation states, and persistent immunological dysfunction in subsets of individuals with post-acute symptoms [5,58,62,104]. These changes may influence pancreatic β-cells indirectly by maintaining systemic inflammation and impairing immune tolerance, while direct adaptive immune-mediated destruction of β-cells remains less clearly demonstrated.

CD8^+^ cytotoxic T lymphocytes are essential for eliminating virus-infected cells but may also contribute to tissue injury when activation is prolonged or inadequately regulated. SARS-CoV-2-specific and bystander CD8^+^ T-cell responses vary according to disease severity, age, antigen persistence, and the quality of immune regulation [105,106]. Persistent cytotoxic activation could intensify tissue and β-cell stress in susceptible individuals. Direct evidence that SARS-CoV-2-specific CD8^+^ T cells selectively target human pancreatic β-cells, however, is currently lacking.

Regulatory T cells are important for maintaining peripheral tolerance and limiting excessive innate and adaptive immune responses. Altered T-cell activation and incomplete immune recovery after COVID-19 could impair inflammatory resolution and facilitate sustained immune activation [104,105,106]. Disturbances in immunoregulatory cytokines may contribute to this process. IL-7 supports lymphocyte survival and homeostatic immune reconstitution, whereas IL-10 limits excessive inflammatory signalling.

Altered circulating concentrations of IL-7 and IL-10 have been reported in individuals who developed metabolic abnormalities after SARS-CoV-2 infection [11]. These observations do not establish a direct causal relationship with β-cell injury. They nevertheless support the hypothesis that impaired immune recovery may maintain inflammatory and metabolic stress and thereby contribute indirectly to β-cell dysfunction.

Adaptive immune dysregulation also involves abnormal B-cell activation and autoantibody production. Broad functional autoantibody responses have been identified during COVID-19, including antibodies directed against cytokines, immune-regulatory proteins, and tissue-associated antigens [107]. Severe infection has also been associated with dysregulated naïve B-cell responses and the emergence of de novo autoreactivity [108]. Autoantibodies against chemokines may persist after infection and have been associated with variation in disease course [109].

These findings do not imply that SARS-CoV-2 routinely causes autoimmune diabetes. Infection may instead act as an environmental accelerator in individuals with pre-existing genetic or immunological susceptibility. It could reveal previously subclinical autoimmunity, intensify an ongoing autoimmune process, or reduce the threshold for loss of tolerance. This interpretation is more consistent with the marked heterogeneity of post-COVID metabolic outcomes than a model of uniform virus-induced autoimmune β-cell destruction.

Several non-mutually exclusive processes have been proposed to explain infection-associated autoreactivity. These include bystander lymphocyte activation, epitope spreading, release of normally sequestered antigens after tissue injury, altered B-cell selection, and prolonged exposure to viral antigens. Molecular mimicry is another possible mechanism, although direct evidence linking specific SARS-CoV-2 epitopes to clinically meaningful autoimmune β-cell destruction remains limited. Post-infectious autoimmunity is therefore more likely to arise from the interaction of multiple immunological abnormalities than from a single dominant pathway [60,104,105,107,108,109].

Host genetic background may modify vulnerability to these immune disturbances. Variation in human leukocyte antigen-mediated peptide presentation can influence autoreactive T-cell responses, whereas inherited differences in interferon and cytokine pathways may alter the magnitude and duration of antiviral immunity. These factors provide a biologically plausible explanation for why autoimmune manifestations arise only in a minority of individuals, although their specific contribution to post-COVID β-cell dysfunction requires further investigation.

Longitudinal immune-profiling studies suggest that post-COVID immune abnormalities involve not only changes in lymphocyte abundance but also altered activation, exhaustion, memory formation, and functional differentiation [104]. Such diversity may generate immunological endotypes with different capacities for viral clearance, inflammatory resolution, tissue repair, and metabolic adaptation. Identifying these endotypes may help distinguish individuals in whom adaptive immunity is a major pathogenic driver from those in whom it primarily reflects persistent viral antigen exposure or systemic inflammation.

Adaptive immune dysregulation should therefore be interpreted as one component of a broader pathogenic network. T-cell activation, impaired regulatory responses, B-cell dysregulation, autoantibody production, and genetic susceptibility interact with viral persistence, endothelial injury, mitochondrial dysfunction, oxidative stress, ER stress, and adipose tissue inflammation.

Important uncertainties remain. Most studies have characterised circulating immune populations rather than immune-cell infiltration or destruction of human pancreatic islets. Longitudinal investigations connecting immune phenotypes with islet autoantibodies, C-peptide dynamics, and direct measures of β-cell function are also scarce. It remains uncertain whether adaptive immune dysregulation is a primary cause of post-COVID β-cell dysfunction, an amplifier of pre-existing susceptibility, or a consequence of persistent viral and inflammatory stimulation.

Overall, available evidence supports adaptive immune dysregulation as a potential contributor to β-cell dysfunction in selected genetically or metabolically susceptible individuals. SARS-CoV-2 appears more likely to disturb immune regulation and facilitate the progression of latent autoimmune tendencies than to produce uniform autoimmune β-cell destruction. Integrated immunophenotyping, autoantibody assessment, HLA analysis, and longitudinal metabolic testing will be required to identify clinically relevant patient subgroups.

### 4.9. Integrated Mechanistic Model of SARS-CoV-2-Induced β-Cell Dysfunction

Post-COVID β-cell dysfunction cannot be adequately explained by a single pathogenic mechanism. The available evidence instead supports a systems-level model in which viral, immune, vascular, metabolic, and intracellular stress pathways interact dynamically to determine β-cell susceptibility and long-term glucose homeostasis. The relative contribution of these pathways is likely to vary according to viral burden or persistence, disease severity, genetic background, immune competence, pre-existing metabolic health, age, adiposity, and environmental exposures [5,58,60,62,64].

These mechanisms should not be interpreted as a simple linear sequence. Direct infection of pancreatic cells may occur in selected individuals, but viral antigens retained within pancreatic or extrapulmonary tissues may also sustain innate and adaptive immune activation. Persistent cytokine signalling promotes ROS production, mitochondrial dysfunction, ER stress, and disturbances in intracellular calcium homeostasis. Damaged mitochondria and stressed ER networks, in turn, release signals that reinforce inflammation and impair insulin biosynthesis and stimulus–secretion coupling.

Endothelial dysfunction adds a microenvironmental component by compromising islet perfusion, vascular barrier integrity, and oxygen delivery. Hypoxia and microvascular injury may further impair mitochondrial ATP production and increase cellular stress. At the systemic level, adipose tissue inflammation promotes insulin resistance, altered adipokine secretion, lipotoxicity, and increased β-cell secretory demand. Adaptive immune dysregulation may additionally prolong inflammatory activation or facilitate autoimmune responses in susceptible individuals.

The interaction of these pathways generates multiple positive feedback loops. Oxidative stress aggravates mitochondrial and ER dysfunction; damaged organelles activate inflammatory sensors; inflammation worsens endothelial and adipose tissue dysfunction; and insulin resistance places additional demands on an already stressed β-cell population. β-cell impairment then contributes to hyperglycaemia and altered lipid metabolism, which further intensify glucotoxic, lipotoxic, and inflammatory injury.

Long-term metabolic abnormalities are therefore likely to emerge when the cumulative burden of viral, inflammatory, vascular, and metabolic stress exceeds the adaptive capacity of pancreatic β-cells. This concept of progressive loss of β-cell resilience may explain why individuals exposed to a similar viral infection can experience markedly different outcomes. These range from complete metabolic recovery to transient dysglycaemia, persistent insulin resistance, progressive secretory impairment, new-onset diabetes, or mixed metabolic phenotypes.

The model also emphasises that β-cells should not be viewed as isolated targets of SARS-CoV-2-associated injury. They function within a complex pancreatic and systemic immunometabolic environment shaped by endothelial cells, pericytes, immune cells, adipose tissue, circulating metabolites, and extracellular signals. Persistent β-cell dysfunction may therefore reflect failure of coordinated tissue and inter-organ homeostasis rather than disruption of a single intracellular pathway.

This systems-level framework has important translational implications. A single biomarker is unlikely to capture the biological complexity of post-COVID metabolic dysfunction. Risk stratification may require integrated assessment of glucose regulation, insulin secretory reserve, insulin resistance, inflammatory activity, endothelial injury, immune dysregulation, oxidative stress, and, where feasible, mitochondrial function. Longitudinal evaluation is particularly important because the dominant mechanism may change over time as acute viral and inflammatory effects give way to persistent metabolic or immune abnormalities.

Therapeutic strategies may similarly need to address more than one pathway. Interventions aimed at improving metabolic control, reducing inflammatory and oxidative stress, restoring endothelial function, supporting mitochondrial and ER homeostasis, and preserving β-cell secretory reserve could be complementary rather than mutually exclusive. Treatment selection will ultimately require identification of mechanistically distinct patient subgroups rather than application of a uniform post-COVID metabolic diagnosis.

Substantial knowledge gaps remain. The relative contribution of direct viral infection, viral persistence, immune-mediated injury, endothelial dysfunction, adipose tissue inflammation, and intracellular stress has not been fully quantified. Heterogeneity across experimental models, tissue studies, and clinical cohorts further limits causal interpretation. Many studies are cross-sectional and cannot distinguish pre-existing abnormalities from changes induced or accelerated by SARS-CoV-2 infection.

Future investigations should integrate longitudinal clinical phenotyping with measures of β-cell function and insulin sensitivity, single-cell transcriptomics, spatial multi-omics, proteomics, metabolomics, immune profiling, and functional analyses of human pancreatic tissue. Such approaches may identify dominant mechanisms within individual patient subgroups and support the development of mechanism-based preventive and therapeutic strategies.

In summary, post-COVID metabolic dysfunction is best conceptualised as a multifactorial systems disorder arising from the disruption of interconnected viral, immune, vascular, mitochondrial, ER, adipose, and β-cell networks. Progressive loss of β-cell resilience provides a unifying framework linking these mechanisms while accounting for the pronounced heterogeneity of post-COVID metabolic phenotypes (Figure 1).

## 5. Clinical Evidence and Phenotypic Spectrum of Post-COVID Metabolic Dysfunction

### 5.1. Defining the Post-COVID Metabolic Phenotype

The mechanistic framework outlined in the preceding section provides the biological basis for interpreting the diverse clinical manifestations of post-COVID metabolic dysfunction. Rather than constituting a single disease entity, this condition encompasses a broad spectrum of abnormalities affecting glucose regulation, insulin sensitivity, pancreatic β-cell function, lipid metabolism, body composition, and systemic energy homeostasis. Variation in clinical presentation likely reflects differences in the relative contributions of viral persistence, immune dysregulation, chronic inflammation, oxidative and mitochondrial stress, endothelial injury, and underlying host susceptibility [4,5,18].

Post-COVID metabolic abnormalities are best regarded as a dynamic continuum rather than a fixed clinical state. Following SARS-CoV-2 infection, individuals may develop transient stress hyperglycaemia, persistent fasting or postprandial dysglycaemia, impaired glucose tolerance, insulin resistance, reduced β-cell secretory capacity, newly diagnosed diabetes mellitus, dyslipidaemia, or overlapping cardiometabolic disturbances. These manifestations may resolve, persist, or evolve over time, indicating that the post-COVID metabolic phenotype comprises several partially overlapping clinical trajectories rather than a uniform disorder [4,9,17,18].

A major diagnostic challenge is determining whether an observed metabolic abnormality was caused by SARS-CoV-2, accelerated by the infection, or merely detected during COVID-19 after having remained clinically silent beforehand. Hyperglycaemia during acute illness may result from systemic inflammation, counter-regulatory hormone release, glucocorticoid exposure, altered nutrition, reduced physical activity, or transient insulin resistance and does not necessarily indicate permanent pancreatic β-cell injury. Conversely, metabolic abnormalities persisting after recovery may reflect incomplete resolution of stress-related changes, sustained insulin resistance, reduced β-cell reserve, or continuing immunometabolic dysregulation [9,13,17,110].

The distinction between newly induced disease and unmasked pre-existing metabolic vulnerability is particularly important in individuals with obesity, visceral adiposity, prediabetes, metabolic syndrome, or a family history of diabetes. In these patients, SARS-CoV-2 infection may act as a metabolic stressor that increases insulin requirements and accelerates progression beyond the compensatory capacity of pancreatic β-cells. By contrast, some individuals without substantial baseline metabolic risk may experience reversible abnormalities that improve as inflammation resolves, glucocorticoids are withdrawn, physical activity resumes, and systemic homeostasis is restored [13,17,110,111,112,113].

Clinical expression is largely determined by the balance between insulin resistance and β-cell dysfunction. Predominant insulin resistance may initially be accompanied by compensatory hyperinsulinaemia, whereas insufficient β-cell adaptation can lead to progressive dysglycaemia and overt diabetes. In other individuals, impaired insulin secretion may be more prominent, particularly when infection accelerates pre-existing β-cell vulnerability or an underlying autoimmune process. Mixed phenotypes are also likely and may change during follow-up, further limiting the usefulness of a single diagnostic label [9,13,112,113].

Post-COVID metabolic disease extends beyond glucose homeostasis. Large observational studies have documented increased risks of dyslipidaemia and cardiovascular sequelae, while broader Long COVID cohorts demonstrate persistent multisystem involvement influenced by baseline metabolic health, lifestyle, acute disease severity, and pre-existing comorbidities [111,114,115,116,117,118]. These observations support a model in which the pancreas, adipose tissue, liver, skeletal muscle, immune system, and vascular endothelium participate in a coordinated disturbance of systemic metabolic regulation.

The temporal course is equally important. Metabolic abnormalities identified during hospitalization should not automatically be classified as persistent post-COVID disease. Repeated assessment after recovery is required to determine whether hyperglycaemia resolves, remains stable, or progresses. Longitudinal evaluation also permits the differentiation of transient stress responses from sustained insulin resistance, progressive β-cell failure, glucocorticoid-associated dysglycaemia, and previously undiagnosed diabetes [9,17,111,112,113].

No standardized diagnostic definition specific to post-COVID metabolic dysfunction has yet been established. Existing Long COVID frameworks primarily define the temporal relationship between infection and persistent symptoms but do not provide dedicated metabolic diagnostic criteria [119]. Clinical assessment must therefore rely on established definitions of prediabetes, diabetes, dyslipidaemia, and metabolic syndrome, combined with documentation of their onset, persistence, or deterioration following SARS-CoV-2 infection.

A comprehensive evaluation should consider glycaemic status, insulin sensitivity, β-cell secretory reserve, lipid profile, body composition, medication exposure, inflammatory activity, and relevant cardiometabolic comorbidities. Where clinically indicated, assessment of C-peptide and islet autoantibodies may help distinguish insulin-resistant, insulin-deficient, and potentially autoimmune phenotypes. Such multidimensional characterization is more informative than attributing all post-infectious dysglycaemia to a single mechanism.

Overall, the post-COVID metabolic phenotype is best conceptualized as a temporally evolving and biologically diverse spectrum shaped by the interaction between infection-related stress and pre-existing host vulnerability. This framework provides the basis for interpreting findings from longitudinal cohorts, electronic health-record studies, and international registries.

### 5.2. Clinical Cohort Studies Supporting the Post-COVID Metabolic Phenotype

Evidence from large healthcare databases, prospective physiological studies, and international registries indicates that SARS-CoV-2 infection is followed by an increased burden of metabolic disease in at least a subset of survivors. The strength of this evidence lies not in a single cohort but in the convergence of epidemiological, clinical, and physiological observations across different populations and study designs (Table 3).

One of the most influential analyses was conducted within the United States Veterans Affairs healthcare system. Xie and colleagues reported an increased 12-month risk of incident diabetes and initiation of glucose-lowering therapy after COVID-19, with the burden rising according to the severity of the acute infection [17]. Subsequent analyses from the same healthcare system demonstrated an increased risk of incident dyslipidaemia, indicating that post-acute metabolic sequelae extend beyond glycaemic abnormalities [114]. Longer follow-up further suggested that the duration and overall burden of post-acute outcomes vary according to the severity of the initial illness [118].

The interpretation of these findings requires consideration of the characteristics of the Veterans Affairs population, including its predominance of older men and relatively high prevalence of baseline cardiometabolic disease. Although extensive adjustment was performed, residual confounding and incomplete identification of previously undiagnosed diabetes cannot be fully excluded. Nevertheless, the large sample size, use of contemporary and historical control groups, and consistent severity gradient provide substantial epidemiological support for an association between COVID-19 and subsequent metabolic risk.

A nationwide electronic health-record analysis of approximately 16 million adults in England provided complementary evidence. Taylor et al. observed an increased incidence of diabetes after SARS-CoV-2 infection and found that vaccination reduced, but did not completely eliminate, the excess post-infectious risk [120]. These findings strengthen the temporal association between infection and newly diagnosed diabetes while suggesting that prevention of severe disease may mitigate part of the subsequent metabolic burden.

Population-based analyses have additionally examined factors modifying the risk of broader post-COVID sequelae. In a prospective UK Biobank study, adherence to a healthier lifestyle was associated with a lower risk of multisystem post-COVID outcomes, hospitalization, and death [115]. Although this study was not designed specifically to measure β-cell dysfunction, it supports the importance of modifiable host characteristics in shaping long-term recovery and indicates that post-COVID outcomes reflect interactions between infection and baseline metabolic health.

Detailed physiological studies provide mechanistic context for the epidemiological findings. Montefusco et al. demonstrated persistent abnormalities in glucose homeostasis, insulin sensitivity, and β-cell function following acute COVID-19 [13]. Such studies are generally smaller than population-based cohorts but offer direct metabolic phenotyping that cannot be obtained from diagnostic codes alone. Their findings support the possibility that newly diagnosed dysglycaemia after COVID-19 may reflect measurable disturbances in insulin action and secretion rather than increased clinical surveillance alone.

Cromer et al. further demonstrated that diabetes first recognized during hospitalization for COVID-19 is clinically heterogeneous. Follow-up revealed that some individuals had persistent diabetes, whereas others experienced substantial glycaemic improvement, consistent with transient stress hyperglycaemia or reversible insulin resistance [112]. This variability reinforces the need to avoid equating all diabetes diagnosed during acute COVID-19 with permanent, directly virus-induced β-cell failure.

The CoviDIAB Registry was established to characterize new-onset diabetes associated with COVID-19 and to clarify its natural history and underlying mechanisms [9]. The registry framework recognizes that this presentation may include previously undiagnosed type 2 diabetes, stress hyperglycaemia, glucocorticoid-associated dysglycaemia, accelerated metabolic disease, direct or indirect β-cell injury, and autoimmune diabetes in susceptible individuals. Its principal contribution is therefore the systematic recognition of phenotypic diversity rather than proof of a single COVID-specific form of diabetes.

Large Long COVID programmes provide an additional clinical context. The NIH RECOVER Initiative has demonstrated marked heterogeneity in post-acute symptoms, organ involvement, and recovery trajectories and has developed research indices for identifying adults with Long COVID [121,122]. These studies do not by themselves establish a distinct post-COVID metabolic syndrome, but they provide an essential platform for evaluating metabolic abnormalities within a well-characterized multisystem condition.

European initiatives, including the ORCHESTRA Consortium, have likewise examined long-term outcomes across hospitalized and non-hospitalized populations [123]. Their principal relevance to metabolic research lies in prospective follow-up, standardized data collection, and assessment of interactions among inflammation, comorbidity, acute disease severity, and persistent organ dysfunction. However, diabetes-specific and β-cell-specific conclusions should be drawn only from dedicated metabolic analyses within these cohorts.

Similarly, prospective Spanish studies have characterized the persistence and heterogeneity of post-COVID symptoms and associated clinical risk factors after hospitalization [124]. Although these data contribute to the broader understanding of Long COVID trajectories, the cited study does not directly establish persistent insulin resistance or β-cell dysfunction. It should therefore be considered contextual evidence for the multisystem post-COVID phenotype rather than a primary metabolic cohort.

Taken together, current clinical evidence supports an association between SARS-CoV-2 infection and an increased risk of newly diagnosed diabetes, dyslipidaemia, and broader cardiometabolic sequelae. However, the magnitude and interpretation of this risk vary according to study design, population characteristics, severity of the acute illness, duration of follow-up, vaccination status, and methods used to exclude pre-existing disease. Epidemiological associations should therefore be integrated with longitudinal biochemical and physiological assessment before causality or a specific mechanism is inferred.

### 5.3. New-Onset Diabetes After COVID-19: Clinical Heterogeneity and Pathophysiological Interpretation

New-onset diabetes has emerged as one of the most consistently reported metabolic sequelae of SARS-CoV-2 infection. Large epidemiological studies have demonstrated an increased incidence of diabetes following COVID-19, particularly after severe disease, although considerable heterogeneity exists regarding the clinical phenotype, timing of onset, and long-term metabolic outcomes [13,17,18,112,113,120]. Rather than representing a single disease entity, diabetes first recognized during or after COVID-19 is increasingly regarded as a heterogeneous clinical syndrome resulting from the interaction between infection-related metabolic stress and individual susceptibility.

As discussed in the preceding sections, SARS-CoV-2 may affect glucose homeostasis through multiple interconnected mechanisms, including transient β-cell dysfunction, systemic inflammation, insulin resistance, endothelial injury, mitochondrial dysfunction, and immune dysregulation. However, current evidence suggests that these mechanisms do not contribute equally in every patient, and irreversible viral β-cell destruction is unlikely to explain most cases of diabetes diagnosed after COVID-19 [47,113,125,126]. Instead, the clinical phenotype appears to reflect the combined effects of acute infection superimposed on pre-existing metabolic vulnerability.

One important consideration is that hyperglycaemia detected during acute COVID-19 does not necessarily indicate permanent diabetes. Severe illness is accompanied by profound inflammatory activation, increased counter-regulatory hormone secretion, acute insulin resistance, and, frequently, glucocorticoid therapy, all of which may transiently impair glucose metabolism. Consequently, some patients who fulfil diagnostic criteria for diabetes during hospitalization subsequently return to normoglycaemia or prediabetes after recovery, indicating that stress hyperglycaemia represents an important component of the observed increase in diabetes incidence [18,112]. Longitudinal reassessment is therefore essential before establishing a diagnosis of persistent diabetes.

For many individuals, COVID-19 is more likely to accelerate or unmask pre-existing metabolic disease than to induce an entirely new pathological process. Patients with obesity, visceral adiposity, insulin resistance, metabolic syndrome, impaired glucose tolerance, or limited β-cell functional reserve may maintain normal glucose homeostasis before infection through compensatory hyperinsulinaemia. The metabolic stress imposed by SARS-CoV-2 infection may exceed this compensatory capacity, resulting in overt hyperglycaemia and clinical recognition of diabetes [110,112,113,120]. In this context, COVID-19 may act as a metabolic stress test that reveals previously compensated abnormalities rather than serving as the sole cause of diabetes.

The contribution of autoimmune mechanisms remains considerably less certain. Although SARS-CoV-2 infection has been associated with persistent immune dysregulation, functional autoantibodies, and de novo autoreactivity [104,107,108,109], current evidence does not support a substantial increase in islet autoimmunity at the population level [127]. Autoimmune diabetes occurring after COVID-19 therefore appears to represent a relatively uncommon phenotype, most likely affecting genetically predisposed individuals in whom viral infection may accelerate, rather than independently initiate, the autoimmune process [9,113,127].

These observations support the concept that diabetes first recognized after COVID-19 encompasses several overlapping clinical phenotypes rather than a distinct virus-specific form of diabetes. Depending on the relative contribution of β-cell dysfunction, insulin resistance, glucocorticoid exposure, pre-existing metabolic abnormalities, and host susceptibility, patients may experience transient stress hyperglycaemia, persistent type 2 diabetes, autoimmune diabetes, or intermediate phenotypes with partial recovery of β-cell function over time [112,113]. This clinical heterogeneity probably explains much of the variability reported across epidemiological studies.

From a practical perspective, evaluation of newly diagnosed diabetes after COVID-19 should extend beyond confirmation of hyperglycaemia. Previous glycaemic status, HbA1c, body weight, cardiometabolic risk factors, medication history, and glucocorticoid exposure should all be considered when interpreting the diagnosis. In selected patients, fasting or stimulated C-peptide together with pancreatic autoantibodies may assist in distinguishing insulin-deficient from insulin-resistant phenotypes and improve diagnostic classification [128,129,130]. Because metabolic abnormalities may evolve after the acute infection, repeated follow-up remains essential for determining whether dysglycaemia resolves, stabilizes, or progresses.

Overall, the available evidence supports a multifactorial interpretation of diabetes first recognized after COVID-19. Rather than representing a novel diabetes subtype with a single underlying mechanism, it should be viewed as the clinical convergence of multiple biological processes acting on a susceptible metabolic background. This integrative framework reconciles the mechanistic evidence presented in Section 4 with the heterogeneous clinical presentations observed in epidemiological and longitudinal studies, while emphasizing the need for individualized assessment and long-term metabolic follow-up. 

### 5.4. Interaction Between Oxidative Stress and Insulin Secretion

Oxidative stress represents a central mechanism linking cellular stress pathways to impaired insulin secretion. Excessive production of reactive oxygen species may damage mitochondrial membranes, impair calcium signalling, disrupt insulin biosynthesis, and compromise insulin granule exocytosis, ultimately leading to reduced β-cell function [12,13].

In post-COVID metabolic disturbances, oxidative stress may persist beyond the acute phase of infection and contribute to long-term metabolic instability. Clinical evidence indicates that markers of oxidative stress are strongly associated with impaired β-cell processing and secretory efficiency, supporting the concept that chronic oxidative stress represents a major determinant of β-cell dysfunction [14].

Furthermore, oxidative stress interacts closely with inflammatory signalling pathways, creating a self-perpetuating cycle of cellular injury and metabolic dysregulation. Activation of redox-sensitive transcription factors promotes inflammatory responses, which in turn further increase ROS generation and metabolic stress. This interaction may accelerate the transition from β-cell compensation to β-cell failure in individuals with persistent metabolic abnormalities.

### 5.5. Risk Modifiers and Individual Susceptibility

The considerable heterogeneity of metabolic outcomes following SARS-CoV-2 infection indicates that COVID-19 alone is insufficient to explain the development of persistent dysglycaemia or diabetes. Although viral infection may trigger metabolic disturbances, the eventual clinical phenotype is largely determined by the interaction between infection-related insults and the individual’s pre-existing metabolic and biological susceptibility [130]. Consequently, post-COVID diabetes should be viewed as the result of a multifactorial risk landscape rather than a uniform consequence of viral infection.

Among the most consistently identified risk modifiers are older age, obesity, visceral adiposity, pre-existing insulin resistance, metabolic syndrome, and established cardiometabolic disease [13,17,18,111,124]. These conditions are characterized by chronic low-grade inflammation, impaired metabolic flexibility, and reduced β-cell functional reserve, thereby lowering the threshold at which additional metabolic stress results in overt hyperglycaemia. Rather than acting independently, these factors appear to amplify the metabolic consequences of SARS-CoV-2 infection.

The severity of the acute infection also represents an important determinant of long-term metabolic outcomes. Individuals requiring hospitalization or intensive care consistently demonstrate a greater subsequent risk of diabetes than those with milder disease [17,18,108,124]. Greater inflammatory burden, prolonged immobilization, nutritional alterations, corticosteroid exposure, and the persistence of systemic immune activation probably contribute collectively to this increased risk. Nevertheless, persistent metabolic abnormalities have also been reported after non-hospitalized infections, suggesting that long-term dysglycaemia cannot be explained solely by the severity of the acute illness [102,124].

Host biological characteristics may further influence individual susceptibility. Genetic background, baseline β-cell reserve, immune responsiveness, and the capacity to recover from metabolic stress are all likely to contribute to the observed inter-individual variability [119,128,130]. Although current evidence does not support routine genetic testing in clinical practice, these observations reinforce the concept that SARS-CoV-2 acts within an already existing biological context rather than creating identical metabolic consequences in every patient.

Vaccination status has also emerged as a potential modifier of long-term metabolic risk. Population-based studies suggest that COVID-19 vaccination may reduce the incidence of post-acute complications, including new-onset diabetes, primarily through attenuation of disease severity rather than complete prevention of infection [120,124]. These findings further support the concept that limiting the magnitude of the acute inflammatory response may influence subsequent metabolic outcomes.

Lifestyle factors before and after infection may additionally affect recovery. Excess adiposity, physical inactivity, unhealthy dietary patterns, and poor cardiometabolic health have all been associated with an increased likelihood of persistent post-COVID manifestations, whereas healthier lifestyle behaviours appear to reduce the overall burden of long COVID [114]. Although direct evidence specifically addressing diabetes remains limited, optimization of modifiable cardiometabolic risk factors is biologically plausible and represents an important component of post-COVID management.

The coexistence of cardiovascular, hepatic, renal, and other metabolic abnormalities further illustrates that diabetes developing after COVID-19 rarely occurs in isolation. Instead, it frequently forms part of a broader multisystem cardiometabolic phenotype characterized by persistent metabolic dysfunction extending beyond glucose homeostasis [112,113,115]. Recognition of this broader context is important because comprehensive cardiometabolic risk assessment may provide greater clinical value than focusing exclusively on glycaemic control.

Overall, current evidence indicates that the development of diabetes after COVID-19 reflects the convergence of viral injury with pre-existing metabolic susceptibility, host biological characteristics, and environmental influences. Rather than identifying a single dominant risk factor, future research should aim to define integrated risk profiles capable of identifying individuals most likely to develop persistent metabolic complications after SARS-CoV-2 infection. Such an approach would facilitate personalized follow-up strategies and more targeted preventive interventions.

### 5.6. Clinical Implications for Risk-Adapted Screening and Long-Term Follow-Up

The growing body of epidemiological and mechanistic evidence indicates that metabolic abnormalities constitute an important component of post-COVID condition and may persist for months or even years after resolution of the acute infection. These observations support a risk-adapted, rather than universal, approach to metabolic assessment after SARS-CoV-2 infection. Individuals with pre-existing cardiometabolic disease, hyperglycaemia during acute illness, severe COVID-19, glucocorticoid exposure, or persistent symptoms compatible with Long COVID are likely to derive the greatest benefit from structured metabolic follow-up [4,5,13,17,18].

Initial evaluation should integrate pre-infection metabolic status, the severity and treatment of acute COVID-19, current clinical symptoms, anthropometric measurements, blood pressure, and conventional cardiometabolic risk factors. Fasting plasma glucose and glycated haemoglobin (HbA1c) represent appropriate first-line investigations, while lipid profile assessment should be incorporated into comprehensive cardiometabolic risk evaluation. Oral glucose tolerance testing (OGTT) may be considered in selected patients with previous stress hyperglycaemia, persistent symptoms, discordant fasting glucose and HbA1c values, or persistent clinical suspicion of impaired glucose tolerance despite inconclusive routine testing [13,17,18,60,123].

Closer surveillance appears particularly appropriate for individuals with obesity, visceral adiposity, prediabetes, metabolic syndrome, established cardiovascular disease, prolonged hospitalization, severe systemic inflammation, or substantial glucocorticoid exposure. Likewise, patients who developed hyperglycaemia during acute COVID-19 should undergo repeat metabolic assessment after recovery, since the initial disturbance may represent transient stress hyperglycaemia, previously unrecognized diabetes, or the early manifestation of persistent metabolic dysfunction [13,17,18].

Persistent fatigue, reduced exercise tolerance, unexplained weight change, recurrent hyperglycaemia, polyuria, or polydipsia during recovery should prompt metabolic evaluation irrespective of the severity of the initial infection. However, endocrine abnormalities should be interpreted within the broader clinical context, as post-COVID condition is a multisystem disorder with diverse clinical manifestations. Accordingly, metabolic assessment should be integrated with evaluation of cardiovascular, inflammatory, and other organ-specific complications when clinically indicated [4,5].

Additional investigations should be individualized according to the predominant clinical phenotype. Measurement of fasting or stimulated C-peptide may help assess endogenous β-cell function when insulin deficiency or atypical diabetes is suspected, whereas pancreatic autoantibody testing should generally be reserved for patients with clinical features suggestive of autoimmune diabetes. Biomarkers of insulin resistance, including fasting insulin and HOMA-IR, together with emerging molecular approaches such as metabolomics, proteomics, transcriptomics, and immune profiling, remain valuable research tools but currently lack sufficient validation for routine clinical management [13,60,119].

The optimal duration and frequency of metabolic surveillance remain uncertain. Current evidence supports periodic reassessment during the first year after infection in individuals at increased metabolic risk, although standardized follow-up protocols have not yet been established. In patients with hyperglycaemia during hospitalization, reassessment approximately three to six months after recovery may help distinguish transient metabolic disturbances from persistent abnormalities and provide a more reliable evaluation of post-acute glycaemic status [17,18]. Thereafter, the frequency of follow-up should be individualized according to baseline metabolic risk, persistence of symptoms, and longitudinal changes in metabolic parameters.

Management should remain phenotype-based rather than COVID-specific. Patients meeting established diagnostic criteria for diabetes or prediabetes should receive evidence-based care according to current international guidelines, including lifestyle intervention, weight management, cardiovascular risk reduction, and pharmacological treatment when indicated. Importantly, normalization of glycaemic parameters should not necessarily preclude continued surveillance in individuals with significant metabolic risk factors, as SARS-CoV-2 infection may have revealed limited β-cell reserve or accelerated progression of underlying metabolic disease.

Future clinical practice is likely to combine conventional metabolic assessment with biomarkers reflecting β-cell stress, insulin resistance, endothelial dysfunction, chronic inflammation, and immune activation. Together with advances in multi-omics technologies and artificial intelligence-assisted risk prediction, these approaches may improve early identification of individuals at increased risk for persistent metabolic complications and facilitate more personalized follow-up strategies. Nevertheless, prospective validation is required before these emerging tools can be incorporated into routine clinical practice [131,132,133,134,135,136].

Overall, the available evidence supports targeted longitudinal metabolic surveillance rather than universal screening after SARS-CoV-2 infection. Recognition of post-COVID metabolic dysfunction as a heterogeneous and potentially progressive condition provides an opportunity for earlier diagnosis, individualized intervention, and more effective prevention of future cardiometabolic complications. As our understanding of the underlying mechanisms continues to evolve, integration of clinical assessment with validated biomarkers may further improve risk stratification and therapeutic decision-making (Table 4).

## 6. Biomarkers for Early Detection and Risk Stratification of Post-COVID Metabolic Dysfunction

### 6.1. Biomarkers of β-Cell Function, Stress, and Injury

Early identification of β-cell dysfunction remains one of the principal challenges in the metabolic follow-up of individuals recovering from SARS-CoV-2 infection. Conventional glycaemic measures—including fasting plasma glucose, oral glucose tolerance testing (OGTT), and glycated haemoglobin (HbA1c)—are appropriate for detecting dysglycaemia but provide limited information regarding the mechanisms responsible for its development. In particular, they do not distinguish between impaired insulin secretion, insulin resistance, reversible β-cell stress, or progressive β-cell injury. Consequently, biomarkers reflecting endogenous insulin secretion, β-cell stress, and functional reserve may improve mechanistic phenotyping and facilitate earlier identification of individuals at increased risk of persistent metabolic dysfunction. Nevertheless, most candidate biomarkers have been validated in type 1 or type 2 diabetes rather than specifically in post-COVID populations [128,129,137,138,139,140,141,142].

Among currently available biomarkers, C-peptide remains the most clinically applicable indicator of endogenous β-cell secretory capacity. Interpretation together with simultaneous plasma glucose—and, when appropriate, stimulated C-peptide testing—provides valuable information regarding residual insulin secretory reserve and helps distinguish insulin-deficient from insulin-resistant phenotypes. However, C-peptide reflects β-cell function rather than direct cellular injury, and its interpretation should always consider glycaemic status, renal function, insulin resistance, and ongoing treatment [128,129].

No single biomarker adequately captures the different biological dimensions of β-cell pathology. Dynamic functional indices, including HOMA-B, the insulinogenic index, disposition index, and proinsulin-based measures, provide complementary information regarding β-cell compensation and secretory stress, but most require standardized metabolic testing and remain primarily research tools rather than routine clinical investigations [137,138,139,140,141]. Likewise, emerging molecular biomarkers—including β-cell-enriched microRNAs, extracellular-vesicle cargo, and β-cell-specific cell-free DNA—offer the potential to detect active cellular stress or injury before overt deterioration of glucose homeostasis. However, their clinical implementation is currently limited by methodological complexity, incomplete standardization, and the absence of prospective validation in post-COVID cohorts [142,143,144,145].

An important conceptual distinction should be maintained between biomarkers of β-cell function, β-cell stress, and β-cell injury, as these processes are biologically related but not interchangeable. Functional markers such as C-peptide primarily estimate endogenous insulin secretory capacity, proinsulin-based measures reflect impaired prohormone processing and secretory stress, whereas cell-free DNA and selected extracellular-vesicle signatures may indicate active cellular injury. Recognition of these complementary biological domains is essential for accurate interpretation of biomarker findings and underscores why reliance on a single marker is unlikely to provide a comprehensive assessment of post-COVID β-cell dysfunction.

Overall, current evidence supports a multimarker approach rather than dependence on any individual biomarker. Conventional glycaemic tests remain appropriate for initial metabolic screening, whereas C-peptide-based assessment and selected dynamic functional indices may provide additional phenotypic information in appropriately selected patients. As emerging molecular biomarkers become standardized and prospectively validated, integration of markers reflecting β-cell function, insulin sensitivity, inflammation, endothelial dysfunction, and immune activation may further improve early risk stratification and enable more personalized management of post-COVID metabolic dysfunction (Table 5).

### 6.2. Inflammatory and Immunological Biomarkers

Persistent low-grade inflammation and immune dysregulation are increasingly recognized as central features of post-COVID metabolic dysfunction. Accordingly, circulating inflammatory and immunological biomarkers may provide complementary information regarding ongoing immune activation, insulin resistance, endothelial dysfunction, β-cell stress, and long-term metabolic risk. However, no single biomarker currently demonstrates sufficient specificity or predictive accuracy for routine risk stratification. Instead, integration of inflammatory, immunological, and metabolic biomarkers is likely to provide greater mechanistic and prognostic value than isolated cytokine measurements [60,141,142,143].

Rather than reflecting distinct pathological pathways, inflammatory biomarkers should be interpreted as components of interconnected immunometabolic networks. Pro-inflammatory cytokines, including IL-6, TNF-α, IL-1β, and IL-18, collectively reflect persistent inflammatory activation that may contribute to insulin resistance, endothelial dysfunction, and impaired β-cell function. Nevertheless, their circulating concentrations are influenced by obesity, acute disease severity, comorbidities, sampling time, and other systemic inflammatory conditions, substantially limiting their specificity for post-COVID metabolic dysfunction [60,146,147,148,149,150,151].

Inflammasome-associated mediators, particularly IL-1β and IL-18, provide indirect evidence of persistent NLRP3 inflammasome activation, a pathway implicated in both acute SARS-CoV-2 infection and chronic metabolic inflammation. While biologically plausible, their prognostic value for predicting long-term β-cell dysfunction or progression to diabetes remains insufficiently validated, and their current role is primarily mechanistic rather than diagnostic [149,150,151].

Routine inflammatory biomarkers, including high-sensitivity C-reactive protein (hsCRP) and ferritin, remain readily available indicators of systemic inflammation. However, because both are strongly influenced by adiposity, liver disease, iron metabolism, infection, and numerous inflammatory conditions, they should be interpreted as markers of overall inflammatory burden rather than direct indicators of pancreatic injury [148,150].

Additional insight into persistent immune dysregulation may be provided by interferon-related mediators, chemokines, and immunoregulatory cytokines. Biomarkers such as IFN-γ, CXCL10/IP-10, CCL2/MCP-1, IL-17A, IL-7, and IL-10 reflect different aspects of adaptive immune activation, immune recovery, and chronic inflammatory signaling. Although these mediators are biologically plausible candidates, their prognostic significance for persistent metabolic dysfunction remains uncertain because prospective longitudinal validation is still lacking [11,146,147,148].

Emerging evidence further suggests that individuals with post-COVID metabolic dysfunction may exhibit cytokine profiles distinct from those observed in conventional metabolic syndrome, supporting the concept of a unique post-COVID immunometabolic phenotype [11]. However, whether these immune signatures represent causal mechanisms, biomarkers of disease activity, or epiphenomena remains unresolved and requires confirmation in prospective studies.

Overall, inflammatory and immunological biomarkers should be regarded as complementary tools for mechanistic phenotyping rather than disease-specific diagnostic markers. Their greatest potential lies in identifying distinct immunometabolic phenotypes and, when integrated with biomarkers of β-cell function, insulin resistance, endothelial dysfunction, and clinical risk factors, improving future strategies for individualized risk stratification and precision medicine (Table 6).

### 6.3. Biomarkers of Oxidative Stress and Mitochondrial Dysfunction

Oxidative stress and mitochondrial dysfunction are increasingly recognized as major contributors to post-COVID metabolic abnormalities and may precede clinically overt impairment of glucose homeostasis. Consequently, biomarkers reflecting redox imbalance and mitochondrial integrity have attracted considerable interest as potential tools for mechanistic phenotyping and early risk stratification. However, despite their biological relevance, most remain investigational and currently complement, rather than replace, conventional metabolic assessment [152,153,154,155].

Rather than relying on a single marker, assessment of oxidative stress should be viewed as a multidimensional process encompassing oxidative damage, antioxidant defence, and mitochondrial homeostasis. Biomarkers such as 8-hydroxy-2′-deoxyguanosine (8-OHdG) and malondialdehyde (MDA) reflect oxidative DNA and lipid damage, whereas endogenous antioxidant systems—including reduced glutathione (GSH), superoxide dismutase (SOD), and glutathione peroxidase (GPx)—provide complementary information regarding the capacity to counteract reactive oxygen species. Although these biomarkers collectively characterize systemic redox status, none is specific for pancreatic β-cell injury or post-COVID metabolic dysfunction [153,154,155,156].

Beyond conventional oxidative stress markers, increasing attention has focused on regulatory pathways that coordinate cellular adaptation to oxidative injury. Nuclear factor erythroid 2-related factor 2 (NFE2L2/NRF2) represents a central regulator of antioxidant defence and mitochondrial homeostasis, linking oxidative stress with cellular resilience. Likewise, biomarkers reflecting mitochondrial integrity—including circulating cell-free mitochondrial DNA (cf-mtDNA), mitochondrial-derived peptides, and metabolomic signatures of mitochondrial bioenergetics—may provide additional insight into persistent cellular stress and impaired oxidative phosphorylation. However, these approaches remain largely confined to research settings because of methodological complexity and limited clinical validation [155,156,157,158].

A major limitation of oxidative stress biomarkers is their lack of disease specificity. Circulating concentrations are influenced by ageing, obesity, cardiovascular disease, chronic inflammation, smoking, and numerous systemic disorders, making isolated interpretation difficult. Consequently, oxidative stress biomarkers are unlikely to serve as independent diagnostic tools but may substantially improve mechanistic characterization when interpreted alongside biomarkers of β-cell function, inflammation, endothelial dysfunction, and insulin resistance.

Overall, biomarkers of oxidative stress and mitochondrial dysfunction should currently be regarded as complementary research tools rather than routine clinical investigations. Their greatest value lies in improving understanding of disease mechanisms and identifying biological pathways associated with persistent metabolic dysfunction. Future multimarker approaches integrating oxidative, inflammatory, mitochondrial, and metabolic biomarkers may enhance early risk stratification and facilitate more precise characterization of post-COVID metabolic phenotypes (Table 7).

### 6.4. Endothelial and Vascular Biomarkers

Persistent endothelial dysfunction is increasingly recognized as a central feature of post-COVID pathophysiology, linking chronic inflammation, microvascular impairment, tissue hypoxia, and metabolic dysregulation. Because pancreatic β-cells depend on an extensive microvascular network for adequate oxygen delivery, nutrient exchange, and efficient insulin secretion, endothelial injury may contribute to persistent impairment of β-cell function. Consequently, circulating biomarkers of endothelial activation may provide complementary information regarding vascular mechanisms underlying post-COVID metabolic dysfunction [82,84,159,160].

Rather than reflecting isolated vascular abnormalities, endothelial biomarkers should be interpreted as indicators of a broader process of vascular inflammation and endothelial activation. Adhesion molecules, including vascular cell adhesion molecule-1 (VCAM-1), intercellular adhesion molecule-1 (ICAM-1), and E-selectin, reflect persistent endothelial activation and leukocyte recruitment, whereas biomarkers such as von Willebrand factor (vWF), angiopoietin-2 (Ang-2), and soluble thrombomodulin provide complementary information regarding endothelial injury, vascular permeability, and disruption of endothelial homeostasis. Collectively, these biomarkers suggest persistent vascular dysfunction that may contribute to impaired tissue perfusion, local hypoxia, insulin resistance, and altered β-cell function following SARS-CoV-2 infection [82,84,159,160,161,162].

Despite their mechanistic relevance, endothelial biomarkers remain limited by low disease specificity. Their circulating concentrations are influenced by ageing, obesity, cardiovascular disease, systemic inflammation, coagulation abnormalities, and numerous chronic disorders, making isolated interpretation challenging. Consequently, these biomarkers are unlikely to become independent diagnostic tools but may provide important complementary information when interpreted together with biomarkers of β-cell function, inflammation, oxidative stress, and metabolic status.

Overall, endothelial and vascular biomarkers provide valuable insight into the vascular component of post-COVID metabolic dysfunction rather than serving as disease-specific diagnostic markers. Their greatest potential lies in improving mechanistic phenotyping and identifying individuals with persistent microvascular injury who may be at increased risk of long-term metabolic complications. Prospective studies are required to determine whether endothelial biomarkers improve risk prediction or represent therapeutic targets capable of modifying long-term cardiometabolic outcomes after SARS-CoV-2 infection (Table 8).

### 6.5. Emerging Multi-Omics Biomarkers

Conventional biomarkers provide valuable information regarding individual biological processes but are inherently limited in capturing the complex molecular interactions underlying post-COVID metabolic dysfunction. Multi-omics technologies overcome this limitation by integrating complementary biological layers—including genomics, transcriptomics, proteomics, metabolomics, lipidomics, and epigenomics—to generate comprehensive molecular signatures that more accurately reflect disease pathophysiology. These approaches have substantially expanded opportunities for biomarker discovery, mechanistic phenotyping, and identification of novel therapeutic targets following SARS-CoV-2 infection [60,131,132,133].

Among currently available platforms, metabolomics and proteomics have emerged as particularly promising approaches for characterizing persistent metabolic dysfunction. Alterations in amino acid metabolism, tricarboxylic acid (TCA) cycle intermediates, acylcarnitines, lipid metabolism, inflammatory pathways, coagulation, extracellular matrix remodeling, and endothelial signalling collectively provide insight into the coordinated molecular networks underlying post-COVID metabolic abnormalities [60,132,134,163]. Rather than serving as individual biomarkers, these molecular signatures offer integrated characterization of disease biology that may precede clinically overt dysglycaemia.

Additional advances in circulating microRNAs, extracellular vesicles, epigenetic profiling, single-cell RNA sequencing, and spatial transcriptomics have further refined understanding of β-cell heterogeneity, mitochondrial dysfunction, inflammatory activation, endoplasmic reticulum stress, and altered intercellular communication following SARS-CoV-2 infection. These technologies continue to identify novel candidate biomarkers while providing unprecedented mechanistic insight into disease pathogenesis [125,126,144,164,165,166].

Despite their considerable promise, most multi-omics technologies remain investigational. Methodological standardization, external validation, reproducibility across populations, and demonstration of clinical utility remain essential before these approaches can be incorporated into routine clinical practice. Nevertheless, by integrating multiple complementary biological domains, multi-omics platforms are expected to play a central role in the future development of precision diagnostics and individualized management strategies for post-COVID metabolic dysfunction (Table 9).

### 6.6. Integrated Biomarker Panels and AI-Based Risk Prediction

The biological complexity of post-COVID metabolic dysfunction cannot be adequately characterized by any single biomarker. Instead, persistent metabolic abnormalities arise through the interaction of β-cell dysfunction, chronic inflammation, oxidative stress, mitochondrial impairment, endothelial injury, immune dysregulation, and systemic metabolic disturbances. Consequently, current research is increasingly shifting from individual biomarkers toward integrated biomarker panels capable of capturing multiple complementary biological processes and providing a more comprehensive assessment of disease risk and progression [60,131,133,134,167].

Integrated biomarker strategies combine conventional metabolic indices with molecular biomarkers and multi-omics signatures to improve mechanistic phenotyping and individualized risk stratification. Rather than relying on isolated laboratory measurements, these multidimensional approaches integrate biomarkers reflecting β-cell function, inflammation, oxidative stress, endothelial dysfunction, mitochondrial biology, and systemic metabolism, thereby providing substantially greater biological resolution than any individual marker alone.

Host genetic susceptibility represents an additional dimension of precision risk assessment. Polygenic risk scores together with variants involved in immune regulation, inflammatory signalling, mitochondrial function, and glucose metabolism may partly explain the marked interindividual variability in metabolic resilience following SARS-CoV-2 infection. Although their current clinical utility remains limited, incorporation of genetic susceptibility into integrated predictive models represents an important future direction for precision medicine [168,169].

The rapid expansion of multidimensional datasets has also accelerated the application of artificial intelligence (AI) and machine-learning approaches. By integrating clinical characteristics, laboratory findings, imaging data, and multi-omics profiles, these computational methods may improve early identification of individuals at risk for persistent β-cell dysfunction, insulin resistance, dysglycaemia, and new-onset diabetes following COVID-19. In addition, AI-driven models may facilitate identification of biologically distinct patient endotypes, enabling more personalized monitoring strategies and optimized clinical trial design [135,136,170].

Overall, integrated biomarker panels combined with AI-assisted analytical approaches represent a promising step toward precision medicine in post-COVID metabolic dysfunction. Nevertheless, analytical standardization, external validation, prospective evaluation in large longitudinal cohorts, demonstration of clinical utility, and cost-effectiveness analyses remain essential before these strategies can be implemented in routine clinical practice (Table 10).

## 7. Therapeutic Perspectives: From Conventional Management to Precision Medicine

### 7.1. Current Therapeutic Approaches

Although no therapy has been specifically approved for post-COVID metabolic dysfunction, current management is largely based on established strategies for diabetes prevention and treatment of metabolic disease. Lifestyle interventions—including dietary modification, regular physical activity, weight reduction, and optimization of cardiovascular risk factors—remain the cornerstone of management. In individuals with persistent dysglycaemia or newly diagnosed diabetes, treatment should follow current international clinical guidelines while recognizing the heterogeneous mechanisms underlying post-COVID metabolic abnormalities [171,172].

At present, there is insufficient evidence to recommend therapeutic strategies that differ from established diabetes management solely on the basis of prior SARS-CoV-2 infection. Consequently, treatment decisions should remain individualized and guided by the patient’s metabolic phenotype, comorbidities, and current international recommendations.

For patients meeting standard indications, metformin remains an appropriate first-line glucose-lowering therapy because, in addition to improving insulin sensitivity, it exerts anti-inflammatory effects and may favorably modulate mitochondrial metabolism [173]. SGLT2 inhibitors provide effective glycaemic control together with well-established cardiovascular and renal protection and may additionally attenuate systemic inflammation, oxidative stress, and mitochondrial dysfunction [174]. GLP-1 receptor agonists (GLP-1RAs) and dual GIP/GLP-1 receptor agonists, including tirzepatide, provide additional benefits through weight reduction, enhancement of glucose-dependent insulin secretion, reduction in β-cell workload, and improvement of surrogate measures of β-cell function [174,175,176,177]. Although direct evidence in post-COVID metabolic dysfunction remains limited, these therapies represent promising candidates for future clinical investigation.

### 7.2. Emerging Mechanism-Based Therapeutic Strategies

Improved understanding of the molecular mechanisms underlying post-COVID metabolic dysfunction has highlighted several potential therapeutic targets extending beyond conventional glucose-lowering therapy. Because persistent inflammation, oxidative stress, mitochondrial dysfunction, endothelial injury, and β-cell stress collectively contribute to disease progression, interventions directed at these pathways may provide additional therapeutic benefit.

Potential approaches include IL-1 antagonists and NLRP3 inflammasome inhibitors to attenuate chronic inflammatory signalling. Therapies targeting oxidative stress have attracted increasing interest, particularly NRF2 activators, which may enhance endogenous antioxidant defence and restore redox homeostasis [178]. Likewise, mitochondria-targeted antioxidants, including compounds such as MitoQ and elamipretide, are being investigated for their ability to preserve mitochondrial function and reduce oxidative injury. Therapeutic strategies targeting cellular senescence, including senolytic and senomorphic agents, have also emerged as promising investigational approaches because senescent cells may contribute to persistent inflammation, tissue remodeling, and β-cell dysfunction following severe systemic injury [179]. Although these therapeutic strategies remain largely investigational, they illustrate the ongoing transition from symptomatic metabolic control toward mechanism-based intervention.

### 7.3. Precision Medicine and Future Therapeutic Directions

Future management of post-COVID metabolic dysfunction will likely rely increasingly on precision medicine, integrating multi-omics biomarkers, clinical phenotyping, and genetic susceptibility to guide individualized therapeutic strategies. Rather than applying a uniform treatment approach, future interventions may be tailored according to the predominant pathogenic mechanisms identified in individual patients, including β-cell dysfunction, insulin resistance, chronic inflammation, mitochondrial impairment, or endothelial injury [131,168,169].

Rapid advances in multi-omics technologies, artificial intelligence (AI), and machine-learning algorithms are expected to improve patient stratification and prediction of therapeutic response by integrating clinical characteristics, laboratory findings, imaging data, wearable-device data, and molecular profiles into multidimensional predictive models [135,136,170].

In parallel, regenerative approaches—including induced pluripotent stem cell (iPSC)-derived β cells, pancreatic organoids, stem-cell-based therapies, and tissue engineering—represent promising investigational strategies aimed at restoring endogenous insulin secretion rather than simply controlling hyperglycaemia [180]. Although these technologies remain at an early stage of clinical development, they exemplify the evolving paradigm of personalized therapy and may ultimately improve long-term metabolic outcomes following SARS-CoV-2 infection.

Collectively, current therapeutic strategies remain largely supportive, whereas future management is expected to increasingly depend on biomarker-guided, mechanism-based interventions targeting the molecular pathways responsible for persistent metabolic dysfunction. Integration of early molecular diagnosis with individualized therapeutic approaches has the potential to shift clinical practice from treatment of established metabolic disease toward prevention of progressive β-cell dysfunction and long-term cardiometabolic complications. Nevertheless, prospective randomized clinical trials, analytical validation of predictive biomarkers, and demonstration of clinical utility remain essential before these strategies can be incorporated into routine clinical practice (Table 11).

## 8. Conclusions and Future Perspectives

The COVID-19 pandemic has substantially advanced our understanding of the complex interplay between viral infection and metabolic disease. Accumulating experimental and clinical evidence demonstrates that SARS-CoV-2 is not merely a respiratory pathogen but a multisystem virus capable of inducing persistent metabolic disturbances that extend well beyond the acute phase of infection. Among these long-term sequelae, pancreatic β-cell dysfunction has emerged as a central mechanism linking SARS-CoV-2 infection to impaired glucose homeostasis, insulin resistance, dysglycaemia, and an increased risk of new-onset diabetes mellitus.

As discussed throughout this review, β-cell dysfunction results from the convergence of multiple interrelated pathogenic mechanisms rather than from a single dominant pathway. Although direct viral infection of pancreatic β-cells has been demonstrated in several experimental studies, its overall contribution remains controversial. Current evidence supports a multifactorial model in which persistent inflammation, immune dysregulation, oxidative stress, mitochondrial dysfunction, endoplasmic reticulum stress, endothelial injury, adipose tissue dysfunction, and altered intercellular communication collectively impair β-cell function and insulin secretion. The dynamic interactions among these processes disrupt glucose sensing, ATP production, calcium signalling, insulin biosynthesis, and insulin granule exocytosis, ultimately reducing the ability of β-cells to maintain metabolic homeostasis.

An important concept emerging from recent studies is that post-COVID metabolic dysfunction should be regarded as a heterogeneous clinical and biological spectrum rather than a single disease entity. Individual susceptibility appears to depend on the complex interplay between pre-existing metabolic health, β-cell functional reserve, genetic background, immune responses, viral persistence, and the capacity to adapt to chronic cellular stress. Consequently, SARS-CoV-2 infection may either precipitate de novo metabolic disease or accelerate the progression of previously subclinical metabolic abnormalities. This heterogeneity explains the wide variability in clinical outcomes, ranging from transient stress-induced dysglycaemia to persistent diabetes requiring long-term treatment.

An equally important concept highlighted throughout this review is that β-cell dysfunction should not be interpreted in isolation but rather within the broader context of systemic metabolic dysregulation. Crosstalk among the pancreas, adipose tissue, the immune system, vascular endothelium, and mitochondria creates a self-perpetuating network of inflammation and metabolic stress that may persist long after viral clearance. Recognition of these interconnected mechanisms provides a more comprehensive framework for understanding the pathogenesis of post-COVID metabolic disease and identifies multiple potential therapeutic targets beyond conventional glucose-lowering strategies.

Despite remarkable progress over recent years, several important questions remain unanswered. The precise contribution of persistent viral reservoirs, autoimmune mechanisms, β-cell dedifferentiation, and mitochondrial dysfunction to long-term metabolic impairment remains incompletely understood. Likewise, reliable biomarkers capable of distinguishing transient post-infectious metabolic disturbances from progressive β-cell failure have not yet been established. Addressing these unresolved issues will require well-designed longitudinal studies integrating molecular, cellular, and clinical data across diverse patient populations.

Future advances will likely be driven by the integration of multi-omics technologies—including genomics, transcriptomics, proteomics, metabolomics, lipidomics, single-cell sequencing, and spatial transcriptomics—together with circulating biomarkers, artificial intelligence, and machine-learning approaches. These rapidly evolving technologies offer unprecedented opportunities to identify biologically distinct patient subgroups, improve early risk stratification, and develop personalized therapeutic strategies based on the predominant molecular mechanisms underlying disease progression. Similarly, mechanism-based interventions targeting chronic inflammation, oxidative stress, mitochondrial dysfunction, endothelial injury, and preservation of β-cell function may represent the next generation of therapies for preventing or delaying post-COVID metabolic complications. Ultimately, translating these mechanistic insights into clinical practice will require prospective longitudinal studies, standardized biomarker validation, and randomized clinical trials evaluating mechanism-based therapeutic strategies.

Collectively, the evidence reviewed in this article supports a paradigm shift from viewing post-COVID metabolic abnormalities as isolated disturbances of glucose regulation toward recognizing them as a complex systems disorder arising from dynamic interactions among pancreatic β-cells, immune responses, mitochondrial homeostasis, endothelial integrity, adipose tissue dysfunction, and systemic metabolism. A deeper understanding of these interconnected mechanisms will not only advance our knowledge of post-COVID disease pathogenesis but will also enable earlier diagnosis, improve risk stratification, and facilitate the development of personalized therapeutic strategies aimed at preserving β-cell function and reducing the long-term cardiometabolic burden associated with SARS-CoV-2 infection.

## Figures and Tables

**Table 1:** Host Receptors, Cofactors, and Proteases Implicated in SARS-CoV-2 Entry into Pancreatic β-Cells: Current Evidence.

Molecule	Physiological or Cellular Function	Proposed Role in SARS-CoV-2 Entry	Evidence Related to Pancreatic β-Cells	Overall Strength of Evidence
ACE2	Membrane carboxypeptidase and regulator of the renin–angiotensin system	Principal receptor mediating viral attachment	Detected in some studies, but abundance and localisation remain controversial	Strong for viral entry; moderate and disputed in β-cells
TMPRSS2	Transmembrane serine protease	Promotes spike priming and plasma membrane fusion	Generally low or inconsistently detected in β-cells	Strong for viral entry; limited in β-cells
NRP1	Cell-surface co-receptor involved in vascular and neuronal signalling	Enhances infectivity following furin-mediated spike cleavage	Possible expression in pancreatic endocrine tissue; functional evidence remains limited	Moderate
Furin	Proprotein convertase	Cleaves the spike protein at the S1/S2 site before entry	Indirect evidence; not specific to β-cells	Moderate
Cathepsins B/L	Endosomal cysteine proteases	Support alternative endosomal spike activation	Biologically plausible, particularly when TMPRSS2 expression is low	Moderate
TMPRSS4	Transmembrane serine protease	May complement TMPRSS2-dependent entry	Limited direct evidence in β-cells	Limited
ADAM17	Metalloproteinase involved in ACE2 shedding	May regulate ACE2 availability and inflammatory signalling	Indirect evidence	Limited
DPP4	Membrane glycoprotein and enzymatic receptor for MERS-CoV	Proposed accessory receptor or cofactor	Inconsistent and unconfirmed	Weak
CD147/Basigin	Transmembrane glycoprotein involved in matrix and immune regulation	Proposed facilitator of viral entry	Highly controversial and unconfirmed	Weak

Abbreviations: ACE2, angiotensin-converting enzyme 2; TMPRSS2, transmembrane serine protease 2; NRP1, neuropilin-1; ADAM17, a disintegrin and metalloproteinase 17; DPP4, dipeptidyl peptidase-4. The overall strength of evidence represents a qualitative assessment of the current literature regarding the role of each molecule in SARS-CoV-2 entry into pancreatic β-cells.

**Table 2:** Mechanisms Linking SARS-CoV-2-Associated Stress to Mitochondrial Dysfunction and β-Cell Failure.

SARS-CoV-2-Associated Mechanism	Mitochondrial Consequence	Functional Impact on β-Cells
Persistent inflammatory signaling	Increased mitochondrial ROS production and respiratory-chain dysfunction	Reduced ATP generation and impaired insulin secretion
NADPH oxidase activation	Amplification of cellular and mitochondrial redox imbalance	Oxidative damage to proteins, lipids, and mitochondrial DNA
Disrupted fusion–fission Dynamics	Fragmented or maladaptive mitochondrial networks	Reduced metabolic amplification and secretory competence
Impaired mitophagy	Accumulation of dysfunctional mitochondria	Increased ROS production and reduced bioenergetic efficiency
Calcium dysregulation	Impaired mitochondrial calcium buffering and permeability transition	Defective stimulus–secretion coupling and activation of apoptosis
Suppression of oxidative Phosphorylation	Reduced ATP production	Impaired glucose-stimulated insulin secretion
Persistent metabolic reprogramming towards glycolysis	Reduced reliance on mitochondrial respiration	Potential energetic insufficiency and impaired β-cell adaptation

**Table 3:** Major Clinical Cohorts and Registries Relevant to Post-COVID Metabolic Outcomes.

Study or Cohort	Study Design and Population	Principal Findings and Relevance
Veterans Affairs diabetes cohort— Xie et al.	National retrospective cohort of more than 180,000 individuals who survived the acute phase of COVID-19	Increased 12-month risk of incident diabetes and initiation of glucose-lowering therapy. Risk increased progressively with acute COVID-19 severity [17].
Veterans Affairs dyslipidaemia cohort—Xu et al.	National retrospective analysis within the United States Veterans Affairs healthcare system	Increased post-acute risk and burden of incident dyslipidaemia, extending the metabolic phenotype beyond disturbances of glucose homeostasis [114].
OpenSAFELY-TPP England cohort— Taylor et al.	Nationwide retrospective cohort of approximately 16 million adults	Increased incidence of diabetes after SARS-CoV-2 infection. Vaccination reduced, but did not entirely eliminate, the excess risk [120].
UK Biobank lifestyle study—Wang et al.	Population-based prospective cohort	A healthier pre-infection lifestyle was associated with lower risks of multisystem post-COVID sequelae, hospitalization, and death, supporting the modifying role of baseline host factors [115].
Montefusco et al.	Prospective physiological study of patients with COVID-19 and post-acute follow-up	Demonstrated abnormalities in glucose homeostasis, insulin sensitivity, and β-cell function, providing physiological support for persistent metabolic dysfunction [13].
Cromer et al.	Longitudinal cohort of hospitalized patients with newly diagnosed or pre-existing diabetes	Demonstrated heterogeneous long-term glycaemic trajectories; diabetes first detected during acute COVID-19 did not invariably persist after recovery [112].
CoviDIAB Registry	International registry of individuals with newly diagnosed diabetes associated with COVID-19	Designed to define the natural history and heterogeneous causes of diabetes identified in relation to COVID-19, including stress hyperglycaemia, previously unrecognized diabetes, treatment-related dysglycaemia, β-cell injury, and autoimmunity [9].
NIH RECOVER Initiative	Large prospective, multicentre longitudinal programme involving individuals with and without Long COVID	Characterized the clinical heterogeneity and multisystem nature of Long COVID and developed research indices for adult case identification; provides a platform for dedicated metabolic analyses [121,122].
ORCHESTRA Consortium	European multicentre longitudinal programme including hospitalized and non-hospitalized individuals	Evaluates long-term clinical and multisystem outcomes and the influence of comorbidities and acute disease severity; metabolic conclusions require dedicated subgroup analyses [123].
Spanish post-COVID cohorts—Fernández-de-Las-Peñas et al.	Prospective follow-up of previously hospitalized patients	Characterized persistent post-COVID symptoms and associated risk factors. Provides contextual evidence for heterogeneous long-term trajectories but is not a dedicated study of β-cell dysfunction or insulin resistance [124].
Ayoubkhani et al.	Retrospective matched cohort of individuals discharged after hospitalization for COVID-19	Demonstrated increased rates of readmission, mortality, and new respiratory, cardiovascular, hepatic, renal, and metabolic diagnoses following hospitalization [111].
Veterans Affairs two-year post-acute outcomes study—Bowe et al.	Large longitudinal healthcare-database cohort with up to two years of follow-up	Showed that the duration and burden of post-acute sequelae vary according to acute disease severity, with some risks persisting beyond the first year [118].

**Table 4:** Proposed Framework for Risk-Adapted Metabolic Assessment and Follow-Up After COVID-19.

Clinical Phenotype	Predominant Mechanisms	Suggested Assessment	Proposed Follow-Up
Hyperglycaemia during acute COVID-19	Systemic inflammation, stress response, glucocorticoid exposure	Pre-COVID glycaemic status, fasting plasma glucose, HbA1c	Repeat metabolic assessment after recovery (approximately 3–6 months), earlier if clinically indicated
Transient stress hyperglycaemia	Counter-regulatory hormone excess, acute insulin resistance	Fasting plasma glucose, HbA1c; OGTT if clinically indicated or diagnostic uncertainty persists	Confirm resolution; continue follow-up according to baseline cardiometabolic risk
Persistent insulin resistance	Chronic inflammation, adipose tissue dysfunction, metabolic syndrome	Fasting plasma glucose, HbA1c, lipid profile, blood pressure, anthropometric assessment	Periodic cardiometabolic reassessment (approximately every 6–12 months in high-risk individuals)
Predominant β-cell dysfunction	Functional β-cell impairment associated with viral injury, mitochondrial dysfunction, and ER stress	HbA1c, fasting or stimulated C-peptide; pancreatic autoantibodies when clinically indicated	Endocrinology referral and individualized metabolic follow-up
New-onset diabetes	Multifactorial pathogenesis	Standard diagnostic work-up according to current diabetes guidelines	Guideline-directed diabetes management with individualized follow-up
Suspected autoimmune diabetes	Autoimmune activation in genetically susceptible individuals	Fasting or stimulated C-peptide, pancreatic autoantibodies, ketone assessment	Specialist endocrine evaluation and appropriate insulin management when indicated
Post-COVID metabolic syndrome	Insulin resistance, endothelial dysfunction, chronic inflammation	Comprehensive cardiometabolic risk assessment	Lifestyle intervention and periodic cardiometabolic follow-up
Persistent Long COVID symptoms without overt dysglycaemia	Multifactorial pathophysiology	Symptom-guided metabolic assessment; exclude alternative causes	Individualized reassessment if symptoms persist or metabolic abnormalities emerge

This framework represents a proposed risk-adapted clinical approach based on currently available evidence and established principles of diabetes care. It should not be interpreted as a validated international guideline. Clinical assessment and follow-up should be individualized according to pre-existing metabolic status, acute disease severity, treatment exposure, and longitudinal clinical evolution.

**Table 5:** Candidate Biomarkers of β-Cell Function, Stress, and Injury in Post-COVID Metabolic Dysfunction.

Biomarker/ Assessment	Biological Dimension	Potential Clinical Interpretation	Principal Limitations	Current Clinical Status
Fasting C-peptide with simultaneous plasma glucose	Endogenous β-cell secretory capacity	Low or inappropriately normal C-peptide during hyperglycaemia suggests reduced β-cell reserve; elevated levels may indicate preserved secretion or compensatory hyperinsulinaemia	Influenced by glycaemia, renal function, insulin resistance, and treatment status	Routine clinical practice
Stimulated C-peptide	Dynamic β-cell secretory reserve	More sensitive assessment of residual β-cell function and secretory reserve	Requires standardized stimulation protocols and timed sampling	Specialized clinical use
Proinsulin concentration	β-Cell secretory stress	Elevated levels suggest impaired proinsulin processing and increased β-cell stress	Assay variability and lack of standardized clinical thresholds	Investigational
Proinsulin-to-insulin or proinsulin-to-C-peptide ratio	Prohormone-processing efficiency	Increased ratios indicate declining β-cell functional integrity and defective insulin processing	Influenced by assay methodology, insulin clearance, and renal function	Investigational
HOMA-B	Basal β-cell function	Estimate of fasting β-cell function and compensatory capacity	Limited precision for individual clinical assessment; influenced by insulin resistance	Research/ Epidemiological studies
Insulinogenic index	Early glucose-stimulated insulin secretion	Reduced values indicate impaired first-phase insulin secretion	Requires standardized OGTT	Research/ Specialized clinical use
Disposition index (oral or intravenous)	β-Cell compensation relative to insulin sensitivity	Best physiological estimate of β-cell compensatory capacity	Requires dynamic metabolic testing; methodology differs across studies	Reference research measure
β-Cell-enriched microRNAs	Cellular stress and altered gene regulation	Potential early marker of β-cell stress and inflammatory injury	Limited tissue specificity, pre-analytical variability, and lack of standardization	Experimental
Extracellular- vesicle cargo	Intercellular communication and cellular stress	May reflect β-cell-derived proteins, lipids, and regulatory RNAs	Isolation methods, tissue origin, and analytical protocols remain insufficiently standardized	Experimental
β-Cell-specific cell-free DNA	Active β-cell injury or cell death	Tissue-specific methylation signatures may indicate ongoing β-cell loss	Low circulating abundance, technically demanding assays, and limited clinical validation	Experimental
Composite multimarker panels	Integrated β-cell phenotype	May improve discrimination between β-cell dysfunction, stress, and active injury while enhancing risk stratification	Require prospective validation, standardization, and external replication	Emerging research approach

No investigational or experimental biomarker has yet been validated for routine diagnosis or prediction of post-COVID β-cell dysfunction. Interpretation of C-peptide should always consider simultaneous plasma glucose, renal function, metabolic phenotype, and ongoing treatment. Dynamic indices are protocol-dependent and should not be directly compared across studies without methodological standardization. Future clinical application is likely to rely on multimarker strategies integrating biomarkers of β-cell function, insulin sensitivity, inflammation, endothelial dysfunction, and immune activation.

**Table 6:** Candidate Inflammatory and Immunological Biomarkers Associated with Post-COVID Metabolic Dysfunction.

Biomarker	Biological Process	Potential Clinical Interpretation	Principal Limitations	Current Clinical Status
IL-6	Systemic Inflammation	Marker of persistent inflammatory activation associated with insulin resistance and endothelial dysfunction	Markedly influenced by obesity, acute illness, and comorbidities	Research
TNF-α	Inflammatory insulin resistance	Associated with impaired insulin signalling and β-cell dysfunction	Limited specificity; strongly influenced by Adiposity	Research
IL-1β	Inflammasome Activation	Reflects inflammatory β-cell stress and metabolic inflammation	Limited prospective Validation	Research
IL-18	NLRP3 inflammasome activation	May indicate persistent inflammasome activity	Limited longitudinal Evidence	Research
hsCRP	Low-grade systemic inflammation	General marker of inflammatory burden	Very low disease Specificity	Routine clinical practice
Ferritin	Inflammation and iron metabolism	May reflect persistent systemic inflammation	Influenced by liver disease, iron metabolism, and acute illness	Routine Clinical practice
IFN-γ	Antiviral and Th1 immune response	Marker of persistent cellular immune activation	Variable findings across studies	Research
CXCL10/IP-10	Chemokine Signaling	Associated with chronic immune activation and leukocyte recruitment	No validated clinical thresholds	Research
CCL2/MCP-1	Monocyte Recruitment	Marker of persistent inflammatory recruitment	Limited disease Specificity	Research
IL-17A	Th17-mediated Inflammation	May reflect persistent tissue inflammation and metabolic dysregulation	Prognostic significance remains uncertain	Research
IL-7	Immune Homeostasis	Marker of T-cell survival and immune recovery	Limited validation in post-COVID cohorts	Research
IL-10	Immunoregulation	Reflects compensatory anti-inflammatory activity	Interpretation depends on clinical context	Research
Composite cytokine/ immune panels	Integrated immunometabolic phenotype	May improve identification of persistent immune activation and metabolic risk	Require prospective validation and standardization	Emerging research approach

No inflammatory or immunological biomarker has yet been validated for routine prediction of post-COVID metabolic dysfunction. Individual cytokines should not be interpreted in isolation because their circulating concentrations are influenced by multiple biological and clinical factors. Future clinical application will likely rely on integrated biomarker panels combining inflammatory, immunological, metabolic, and endothelial markers to improve mechanistic phenotyping and individualized risk stratification.

**Table 7:** Candidate Biomarkers of Oxidative Stress and Mitochondrial Dysfunction in Post-COVID Metabolic Dysfunction.

Biomarker	Biological Process	Potential Clinical Interpretation	Principal Limitations	Current Clinical Status
8-Hydroxy-2′- deoxyguanosine (8-OHdG)	Oxidative DNA damage	Marker of persistent ROS-mediated cellular injury	Influenced by systemic oxidative stress; notβ-cell specific	Investigational
Malondialdehyde (MDA)	Lipid peroxidation	Reflects oxidative membrane injury and systemic lipid peroxidation	Limited disease specificity	Investigational
Reduced glutathione (GSH)	Antioxidant Defence	Reduced levels indicate impaired cellular redox capacity	Influenced by nutritional status and systemic disease	Research
Superoxide dismutase (SOD)	Antioxidant enzyme activity	Reduced activity suggests impaired detoxification of reactive oxygen species	Nonspecific marker of oxidative stress	Research
Glutathione peroxidase (GPx)	Antioxidant enzyme activity	Reduced antioxidant defence Capacity	Influenced by selenium status and systemic Disease	Research
NFE2L2/NRF2	Regulation of antioxidant response	Reflects impaired antioxidant defence and mitochondrial resilience	No validated circulating assay for routine clinical use	Experimental
Cell-free mitochondrial DNA (cf-mtDNA)	Mitochondrial Injury	Marker of cellular stress and mitochondrial damage	Not tissue specific influenced by systemic injury	Experimental
Mitochondrial metabolomic signatures	Bioenergetic dysfunction	May identify impaired oxidative phosphorylation and altered mitochondrial metabolism	Limited standardization and complex analytical methods	Experimental
Mitochondrial- derived peptides	Mitochondrial Signalling	Potential marker of mitochondrial adaptation and cellular stress	Early-stage clinical evidence with limited Validation	Experimental
Integrated oxidative stress panels	Global oxidative and mitochondrial phenotype	May improve mechanistic phenotyping and identification of persistent metabolic vulnerability	Require prospective validation and methodological standardization	Emerging research approach

Currently, no biomarker of oxidative stress or mitochondrial dysfunction has been validated for routine diagnosis or prediction of post-COVID metabolic dysfunction. Individual biomarkers should be interpreted within the broader clinical context because oxidative stress is influenced by ageing, obesity, cardiovascular disease, systemic inflammation, and other comorbidities. Future clinical application is likely to rely on integrated multimarker strategies combining oxidative stress biomarkers with measures of β-cell function, inflammation, endothelial dysfunction, mitochondrial integrity, and metabolic status.

**Table 8:** Candidate Endothelial and Vascular Biomarkers in Post-COVID Metabolic Dysfunction.

Biomarker	Biological Dimension	Potential Clinical Interpretation	Principal Limitations	Current Clinical Status
VCAM-1	Endothelial Activation	Marker of persistent vascular inflammation and leukocyte recruitment	Not organ specific; influenced by systemic inflammation	Investigational
ICAM-1	Leukocyte adhesion and endothelial Activation	Reflects endothelial activation and microvascular dysfunction	Limited disease specificity	Investigational
E-selectin	Endothelial activation	Marker of persistent endothelial injury and vascular Inflammation	Influenced by obesity and inflammatory disorders	Investigational
von Willebrand factor (vWF)	Endothelial injury and coagulation	Marker of endotheliopathy and microvascular injury	Affected by coagulation disorders and acute inflammation	Research
Angiopoietin-2 (Ang-2)	Vascular permeability and endothelial destabilization	Reflects ongoing endothelial dysfunction and vascular Remodeling	Limited disease specificity	Research
Soluble thrombomodulin	Endothelial injury	Indicates loss of endothelial Integrity	Limited clinical validation	Research
Integrated endothelial biomarker panels	Global endothelial Phenotype	May improve identification of persistent endothelial dysfunction and vascular risk	Require prospective validation and methodological standardization	Emerging research approach

Currently, no endothelial biomarker has been validated for routine diagnosis or prediction of post-COVID metabolic dysfunction. Individual biomarkers should be interpreted within the broader clinical context because endothelial function is influenced by cardiovascular disease, obesity, ageing, systemic inflammation, coagulation abnormalities, and other comorbidities. Future clinical application will likely rely on integrated biomarker panels combining markers of endothelial injury with biomarkers of β-cell function, inflammation, oxidative stress, and metabolic status to improve mechanistic phenotyping and individualized risk stratification.

**Table 9:** Emerging Multi-Omics Approaches for Biomarker Discovery in Post-COVID Metabolic Dysfunction.

Omics Platform	Biological Dimension	Potential Clinical Application	Current Clinical Status	Omics Platform
Genomics	Genetic susceptibility	Identification of inherited metabolic risk	Research	Genomics
Epigenomics	DNA methylation and histone modifications	Assessment of long-term metabolic reprogramming	Experimental	Epigenomics
Transcriptomics	Global gene-expression Profiling	Mechanistic phenotyping and pathway identification	Research	Transcriptomics
Single-cell RNA sequencing	Cell-specific transcriptional Signatures	Characterization of cellular heterogeneity and target discovery	Experimental	Single-cell RNA sequencing
Spatial transcriptomics	Tissue-specific gene Expression	Spatial mapping of molecular alterations	Experimental	Spatial transcriptomics
Proteomics	Protein expression and signalling networks	Identification of pathogenic pathways and therapeutic targets	Investigational	Proteomics
Metabolomics	Metabolic pathway alterations	Early identification of metabolic dysregulation	Investigational	Metabolomics
Lipidomics	Lipid metabolism and membrane remodeling	Characterization of metabolic phenotype	Investigational	Lipidomics
Circulating microRNAs	Post-transcriptional gene Regulation	Early detection of β-cell stress and metabolic Dysfunction	Experimental	Circulating microRNAs
Extracellular vesicles	Intercellular molecular communication	Liquid biopsy of tissue-specific injury	Experimental	Extracellular vesicles
Integrated multi-omics platforms	Multidimensional molecular phenotyping	Precision biomarker discovery and individualized risk stratification	Emerging research approach	Integrated multi-omics platforms

Most multi-omics technologies remain investigational and currently serve primarily as platforms for biomarker discovery and mechanistic research. Methodological standardization, external validation, and demonstration of clinical utility remain essential before their implementation in routine clinical practice.

**Table 10:** Integrated Biomarker Strategies for Precision Risk Assessment in Post-COVID Metabolic Dysfunction.

Biomarker Class	Representative Biomarkers	Biological Dimension	Potential Clinical Application	Current Clinical Status
β-cell biomarkers	C-peptide, HOMA-B, proinsulin-based indices, disposition index	β-cell function and secretory reserve	Assessment of β-cell dysfunction	Clinical/ Research
Inflammatory biomarkers	IL-6, TNF-α, IL-1β, hsCRP, ferritin	Chronic inflammation and immune activation	Identification of Persistent Inflammatory phenotypes	Clinical/ Investigational
Oxidative stress biomarkers	8-OHdG, MDA, GSH, SOD, GPx	Oxidative stress and mitochondrial dysfunction	Assessment of redox imbalance	Research
Endothelial biomarkers	VCAM-1, ICAM-1, E-selectin, vWF, Ang-2	Endothelial activation and microvascular injury	Evaluation of vascular dysfunction	Investigational
Multi-omics biomarkers	Metabolomics, proteomics, transcriptomics, miRNAs, extracellular vesicles	Integrated molecular phenotyping	Mechanistic characterization and biomarker discovery	Experimental
Genetic biomarkers	Polygenic risk scores, susceptibility loci	Genetic predisposition	Individualized risk assessment	Emerging
AI-assisted computational models	Machine-learning algorithms and multimodal predictive models	Multidimensional data Integration	Personalized risk prediction and clinical decision support	Emerging

Integrated biomarker panels combine complementary molecular and clinical information to improve risk stratification beyond single biomarkers. Although advances in multi-omics technologies, host genetics, and artificial intelligence have substantially expanded opportunities for precision medicine, prospective validation, methodological standardization, demonstration of clinical utility, and cost-effectiveness analyses remain essential before widespread implementation in routine clinical practice.

**Table 11:** Current and Emerging Mechanism-Based Therapeutic Strategies for Post-COVID Metabolic Dysfunction.

Therapeutic Target	Representative Therapies	Potential Mechanisms	Current Status
Insulin resistance	Metformin, SGLT2 inhibitors	Improved insulin sensitivity, modulation of inflammation and mitochondrial metabolism, cardiometabolic protection	Clinical practice
β-cell dysfunction	GLP-1 receptor agonists, dual GIP/GLP-1 receptor agonists (tirzepatide)	Enhanced glucose-dependent insulin secretion, reduced β-cell workload, weight reduction	Clinical practice
Chronic inflammation	IL-1 antagonists, NLRP3 inflammasome Inhibitors	Suppression of chronic inflammatory signalling	Experimental
Oxidative stress and mitochondrial dysfunction	NRF2 activators, mitochondria-targeted antioxidants	Restoration of redox homeostasis and preservation of mitochondrial function	Experimental
Cellular senescence	Senolytic and senomorphic agents	Reduction in senescence-associated inflammation and tissue dysfunction	Experimental
β-cell regeneration	Stem-cell-based therapies, pancreatic organoids, tissue engineering	Restoration of β-cell mass and endogenous insulin secretion	Early clinical development
Precision medicine	Multi-omics profiling, AI-assisted therapeutic Models	Individualized risk stratification and personalized treatment selection	Translational research

Currently, no therapy has been specifically approved for post-COVID metabolic dysfunction. Existing pharmacological interventions should be prescribed according to established indications and current international clinical guidelines. Emerging mechanism-based therapies remain investigational and require prospective clinical validation.

## Data Availability

No new data were created or analyzed in this study. Data sharing is not applicable to this article.

## Abbreviations

The following abbreviations are used in this manuscript:

ACE2	Angiotensin-Converting Enzyme 2
ATP	Adenosine Triphosphate
COVID-19	Coronavirus Disease 2019
ER	Endoplasmic Reticulum
GIP	Glucose-Dependent Insulinotropic Polypeptide
GLP-1	Glucagon-Like Peptide-1
GLUT	Glucose Transporter
HbA1c	Glycated Hemoglobin
HIF-1α	Hypoxia-Inducible Factor-1 Alpha
HOMA-IR	Homeostatic Model Assessment of Insulin Resistance
IFN-γ	Interferon Gamma
IL-6	Interleukin-6
KATP	ATP-Sensitive Potassium Channel
NRP1	Neuropilin-1
OGTT	Oral Glucose Tolerance Test
ROS	Reactive Oxygen Species
SARS-CoV-2	Severe Acute Respiratory Syndrome Coronavirus 2
SNARE	Soluble N-Ethylmaleimide-Sensitive Factor Attachment Protein Receptor
SUR1	Sulfonylurea Receptor 1
TMPRSS2	Transmembrane Serine Protease 2
TNF-α	Tumor Necrosis Factor Alpha
TyG	Triglyceride–Glucose Index
UPR	Unfolded Protein Response
VAMP2	Vesicle-Associated Membrane Protein 2
